# Global burden of tuberculous meningitis in children aged 0–14 years in 2019: a mathematical modelling study

**DOI:** 10.1016/S2214-109X(24)00383-8

**Published:** 2025-01

**Authors:** Karen du Preez, Helen E Jenkins, Leonardo Martinez, Silvia S Chiang, Sicelo S Dlamini, Mariia Dolynska, Andrii Aleksandrin, Julia Kobe, Stephen M Graham, Anneke C Hesseling, Jeffrey R Starke, James A Seddon, Peter J Dodd

**Affiliations:** Desmond Tutu Tuberculosis Centre, Department of Paediatrics and Child Health, Stellenbosch University, Cape Town, South Africa (K du Preez PhD, Prof A C Hesseling PhD, Prof J A Seddon PhD); Department of Biostatistics (H E Jenkins PhD, J Kobe MA) and Department of Epidemiology (L Martinez PhD), School of Public Health, Boston University, Boston, MA, USA; Division of Pediatric Infectious Diseases, Department of Pediatrics, Alpert Medical School, Brown University, Providence, RI, USA (S S Chiang MD); Center for International Health Research, Rhode Island Hospital, Providence, RI, USA (S S Chiang); Research, Information Monitoring, Evaluation, and Surveillance of the National Tuberculosis Management Cluster, Department of Health, Pretoria, South Africa (S S Dlamini MPH); Infection Control in Ukraine, Kyiv, Ukraine (M Dolynska PhD, A Aleksandrin PhD); Department of Paediatrics and Murdoch Children’s Research Institute, Royal Children’s Hospital, University of Melbourne, Melbourne, VIC, Australia (Prof S M Graham PhD); Burnet Institute, Melbourne, VIC, Australia (Prof S M Graham); Division of Infectious Diseases, Department of Pediatrics, Baylor College of Medicine, Houston, TX, USA (Prof J R Starke MD); Department of Infectious Disease, Imperial College London, London, UK (Prof J A Seddon); Sheffield Centre for Health and Related Research, School of Medicine and Population Health, University of Sheffield, Sheffield, UK (Prof P J Dodd PhD)

## Abstract

**Background:**

Tuberculous meningitis is fatal if untreated and can lead to lifelong neurological sequelae. However, to our knowledge, there are no data on the number of children affected by this disease. We aimed to estimate the global disease burden and attributable mortality of childhood tuberculous meningitis by WHO regions, age groups, treatment status, and HIV status in 2019.

**Methods:**

We developed a Bayesian mathematical model to estimate the number of children aged 0–14 years who developed tuberculous meningitis, died from tuberculous meningitis, and did not die from tuberculous meningitis but had neurological sequelae in 2019. We reviewed the literature and used meta-analyses to quantify key parameters used as model inputs: risk of tuberculous meningitis after *Mycobacterium tuberculosis* infection, tuberculous meningitis as a proportion of tuberculosis notification data (ie, routine surveillance data that countries report to WHO), and risk ratios for tuberculous-meningitis mortality by age group. We identified routine tuberculosis surveillance data from countries and literature that reported the proportion of notified childhood tuberculosis that was due to tuberculous meningitis. Country-level data were from Brazil; the USA; Ukraine; South Africa; and the European Centre for Disease Prevention and Control, which included 29 countries but was aggregated and considered as one site. We assumed tuberculosis notification was synonymous with detection and treatment, combined age-disaggregated risk ratios and published meta-analytic estimates of the case-fatality rate in children who received treatment to produce estimates of tuberculous-meningitis mortality by age group and HIV status, and assumed that untreated tuberculous meningitis was always fatal. We assumed similar age-disaggregated risk ratios for neurological sequelae among children who had treatment for tuberculous meningitis and lived as for children who died.

**Findings:**

An estimated 24 000 (95% credible interval 22 300–25 700) children younger than 15 years developed tuberculous meningitis in 2019. Of these children, 13 000 (12 100–13 900) were estimated to have been diagnosed and treated for tuberculous meningitis. Most untreated children were younger than 5 years. Among the 24 000 children with tuberculous meningitis, 16 100 (14 900–17 300) were estimated to have died in 2019, of whom 1101 (6·8%) had HIV. 13 380 (83·1%) of 16 100 deaths were estimated to be in children younger than 5 years and 11 000 (68·3%) were estimated to be in children who did not receive tuberculous-meningitis treatment. Of the 7900 (5800–10 000) children who did not die, 5550 (5110–5980) were estimated to have neurological sequelae.

**Interpretation:**

Our estimates of tuberculous meningitis in children younger than 15 years showed substantial mortality and morbidity. Improved diagnostics and strong health-care systems to facilitate early diagnosis are crucial to improve outcomes, and tuberculosis prevention should be a public health priority.

## Introduction

In 2022, an estimated 1·25 million children younger than 15 years developed tuberculosis and an estimated 214 000 children, most younger than 5 years, died from the disease globally.^1^ Tuberculosis thus remains one of the top 10 causes of mortality in children younger than 5 years globally.^2^ Due to immaturity across the host immune system, including impaired innate immune-cell function, poor antigen presentation, and evolving adaptive responses, children younger than 5 years are more likely than other age groups to develop disseminated forms of disease, including tuberculous meningitis.^3,4^

Tuberculous meningitis is fatal without treatment and, even when treated, mortality and morbidity are high. A systematic review and meta-analysis of 1636 children younger than 15 years with tuberculous meningitis showed that, of those treated, 19·3% (95% CI 14·0–26·1) died and 53·9% (42·6–64·9) of those who did not die developed neurological sequelae.^5^ Children who do not die from tuberculous meningitis often require lifelong physical and medical care, as well as educational and psychological support.^6^ The capacity of health-care services in tuberculosis-endemic countries to address these needs is restricted, and families have substantial caregiving and financial burdens.^7^

Use of routine tuberculosis surveillance data to estimate morbidity and mortality of tuberculous meningitis at a national or global level is challenging due to poor recording and reporting.^8^ Most countries do not use reporting systems that disaggregate cases by specific disease site and, even in countries where a diagnosis of tuberculous meningitis is recorded, data on the clinical staging at diagnosis are often absent. Diagnosis of tuberculous meningitis in the most advanced clinical stage of disease (stage 3) is associated with increased risk of mortality and neurological disability,^5^ making staging of disease important to understanding its disease burden. Deaths from tuberculous meningitis might also go unrecorded due to missed diagnoses or because children die before accessing health-care services.^9^

A complete understanding of the burden of tuberculous meningitis will enhance our ability to focus global tuberculosis-control resources where they are needed. We aimed to estimate the global disease burden and attributable mortality of childhood tuberculous meningitis by WHO regions, age groups, treatment status, and HIV status in 2019.

## Methods

**Figure 1:**
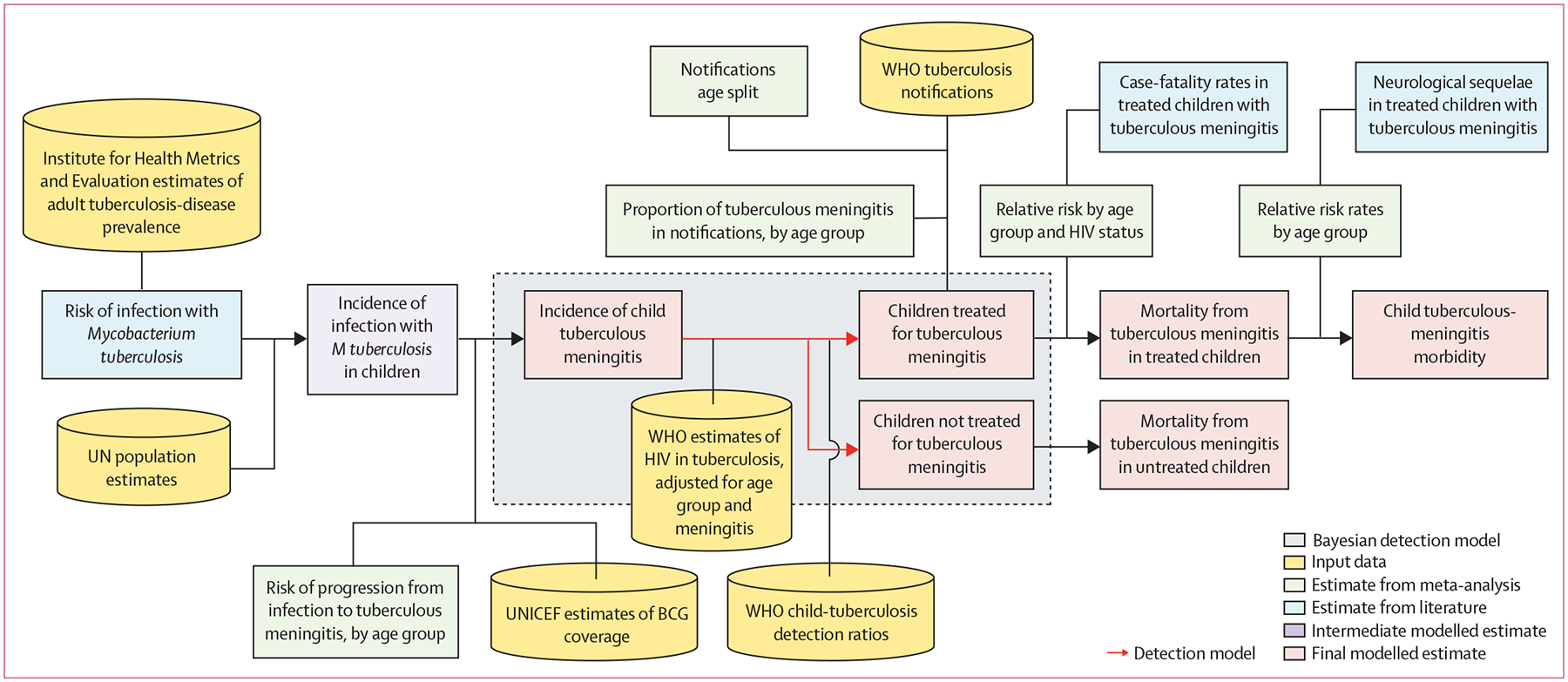
Conceptual framework of our model

**Figure 2:**
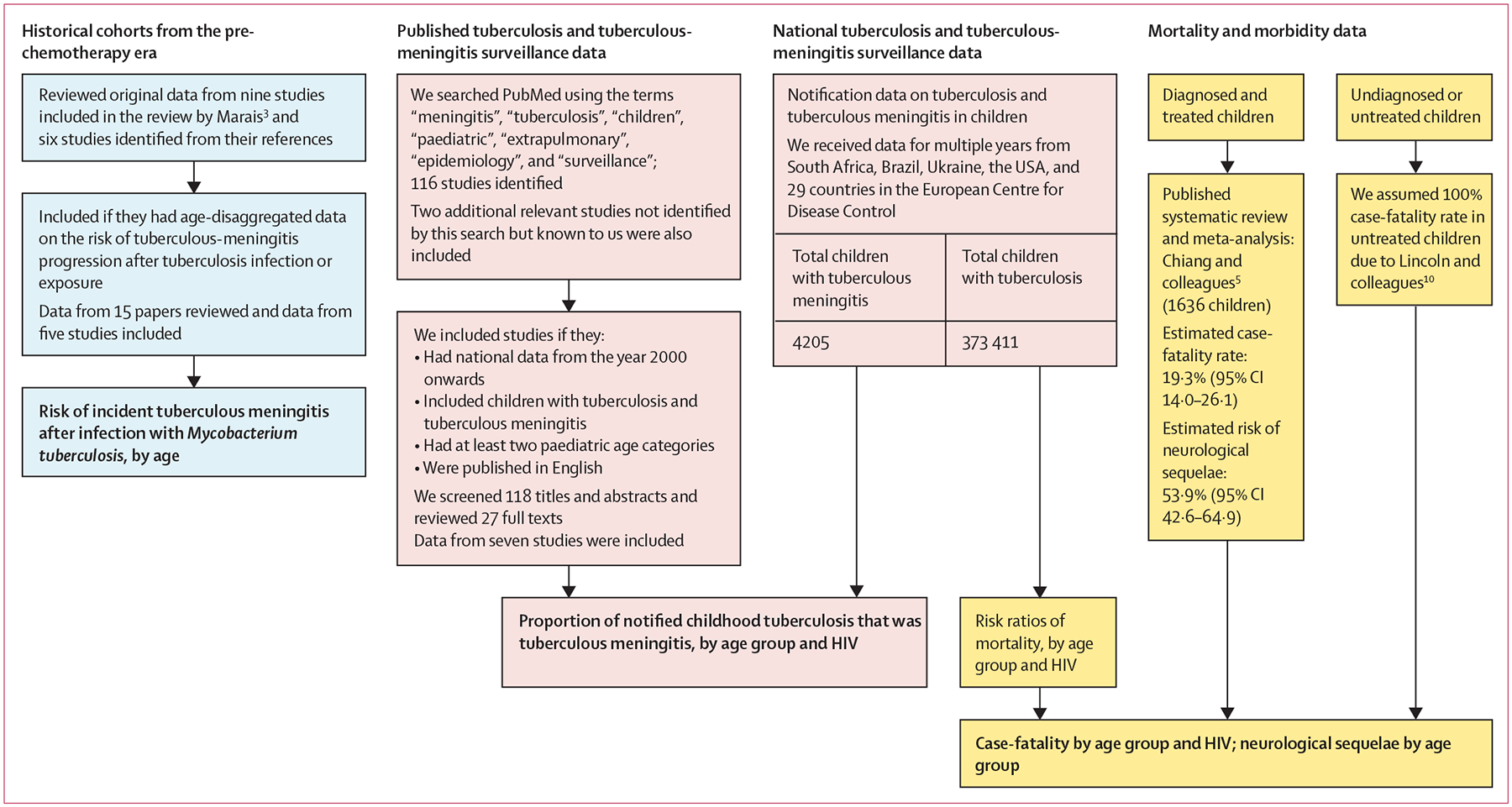
Processes and data sources to inform model parameters References are shown in the appendix (pp 3, 5, 7, 23). Outputs used for the model are shown in bold.

We developed a Bayesian mathematical model (figure 1) to estimate the number of children aged 0–14 years who developed tuberculous meningitis, died from tuberculous meningitis, and did not die from tuberculous meningitis but had neurological sequelae in 2019. We reviewed the literature and used meta-analyses to quantify key parameters used as model inputs: risk of tuberculous meningitis after *Mycobacterium tuberculosis* infection, tuberculous meningitis as a proportion of tuberculosis notification data (ie, routine surveillance data that countries report to WHO), and risk ratios for tuberculous-meningitis mortality by age group and HIV status (figure 2). Our methods and results are reported according to GATHER.^11^

This study was approved by the Stellenbosch University Health Research Ethics Committee (N20/11/042). The study protocol was submitted to the South African National Health Research database (NDOH_202012_002) and approved by the European Centre for Disease Prevention and Control (ECDC).

### Risk of incident tuberculous meningitis after *M tuberculosis* infection

Natural history studies from the pre-chemotherapy era (ie, before 1950) quantified the risk of tuberculous meningitis after *M tuberculosis* infection, stratified by age group. We used a review of the pre-chemotherapy literature^3^ to identify potential studies for inclusion. KdP, HEJ, LM, and JAS sourced and reviewed original papers, and references from these papers, to identify studies that included a defined number of children with evidence of recent *M tuberculosis* infection and that reported tuberculous-meningitis incidence by age group. These authors then extracted data from eligible studies and used random-effects meta-regression against age to estimate the risk of disease progression to tuberculous meningitis in four age groups (ie, <1 year, 1–4 years, 5–9 years, and 10–14 years; appendix p 2).

### Tuberculous meningitis as a proportion of tuberculosis notification data

First, we identified published routine surveillance and programmatic data that reported the number of children with tuberculosis and the number of children with tuberculous meningitis disaggregated by age group (figure 2). We searched PubMed for relevant studies using the search terms “meningitis”, “tuberculosis”, “children”, “paediatric”, “extrapulmonary”, “epidemiology”, and “surveillance” (appendix p 5). Any additional relevant studies that were not identified by this search but were known to any of the authors were also included (appendix p 5).

Second, we obtained routine tuberculosis surveillance data from countries that reported the proportion of notified childhood tuberculosis that was due to tuberculous meningitis, by age group (figure 2). We obtained data from Brazil; the USA; Ukraine; South Africa; and the ECDC, which included 29 countries but was aggregated and considered as one site (appendix p 8).

Our estimates by region were guided by tuberculosis-prevalence estimates from the Institute For Health Metrics and Evaluation^12^ and WHO tuberculosis notifications^1^ as model input data. Country surveillance datasets were only used to generate meta-analytic estimates of the expected proportion of tuberculous meningitis in tuberculosis notifications by age group, and not used to estimate geographical variation.

We used a two-step approach to generate meta-analytic estimates of the proportion of tuberculosis notifications that were due to tuberculous meningitis. First, PJD conducted a meta-analysis of published data and each country-level dataset. For published data, PJD used a random-effects meta-analysis across studies. For country-level data, PJD used a random-effects binomial meta-analysis over years, with data restricted to people without HIV and aggregated by age group. We then conducted a random-effects analysis of estimates from both previous meta-analyses.

### Modelling of tuberculous-meningitis incidence by age group, HIV status, and treatment status

Our Bayesian mathematical model contained priors on tuberculous-meningitis incidence after *M tuberculosis* infection and tuberculous meningitis as a proportion of tuberculosis notifications based on results of our own meta-analyses. We constructed a prior for incidence of *M tuberculosis* infection by applying Styblo’s rule (ie, approximating the annual rate of tuberculosis infection to be proportional to tuberculosis disease prevalence) to tuberculosis-prevalence estimates from the Institute of Health Metrics and Evaluation.^12^ National population size was obtained from UN Population Division demographic estimates.^13^ The prior of risk of incident tuberculous meningitis after *M tuberculosis* infection (obtained from our own meta-analysis) accounted for BCG vaccination coverage and latitude-dependent protection due to varying prevalence of environmental mycobacteria.^14,15^

We included HIV status by disaggregating tuberculous-meningitis incidence. To do this, we scaled the all-age prevalence of HIV in cases of incident tuberculosis for each country estimated by WHO^1^ to a value for children on the basis of a previously published analysis (appendix p 15).^16^ Priors for country-specific tuberculosis case-detection ratios for children aged 0–4 years and aged 5–14 years were based on WHO burden estimates across 202 countries in 2019.^1^ To capture increased risks of tuberculous meningitis and tuberculous meningitis-related death among children with HIV, we applied meta-analytic odds ratios to baseline values on the basis of country-level surveillance data from South Africa (appendix pp 11, 13). Data from South Africa were the only country-level dataset that included notifications and outcomes stratified by HIV status with adequate numbers to include in our meta-analysis to establish tuberculous meningitis as a proportion of tuberculosis notifications and deaths in children living with HIV. We used 2019 WHO tuberculosis notification data^1^ for children aged 0–14 years from all countries and applied meta-analytic estimates of notification age splits to disaggregate notification data into the four age groups included in the model (appendix p 14).

We treated all model parameters as uncertain random variables and constructed a likelihood to relate the estimated tuberculous-meningitis incidence to notified cases of tuberculous meningitis given the WHO estimated case-detection ratio^1^ for each country and by age group. We implemented the model in Stan version 2.32.6 and simulated four chains of 5000 iterations, with the first halves used to adapt and equilibrate the sampling algorithm. We used four chains to generate an increased number of samples (appendix pp 16–18).

The validity of the model was not tested or quantified.

### Modelling tuberculous-meningitis mortality and morbidity by age group and HIV status

Estimates for mortality and morbidity for children aged 0–14 years receiving treatment for tuberculous meningitis were obtained from a published systematic review and meta-analysis.^5^ Mortality was defined as all-cause mortality until treatment completion and neurological sequelae were defined as any neurological impairment that emerged during the illness and persisted through treatment completion, including motor, sensory, cognitive, or hypothalamic involvement.^5^ Age disaggregation of outcome data of the original studies was poor. Few studies that contributed to the systematic review by Chiang and colleagues^5^ reported age-disaggregated outcome data, and their estimates for mortality and neurological sequelae were therefore only reported for children aggregated into one age group: 0–14 years. Therefore, we used data from South Africa to model age distribution of outcomes. In the absence of treatment, we assumed a 100% case-fatality rate on the basis of the pre-chemotherapy literature.^10^

To calculate risk of mortality on treatment by age group and HIV status, we estimated risk ratios from the South African surveillance dataset as it was the only data source with age group and HIV status disaggregated treatment outcome data for tuberculous meningitis. As disease severity is associated with mortality and morbidity, and we expected a similar age distribution, we therefore assumed these risk ratios of mortality by age group also applied to morbidity (ie, neurological sequelae). Risk ratios were not used to estimate overall mortality or morbidity, only to model the age distribution of outcomes estimates.

We assumed tuberculosis notification was synonymous with detection and treatment. We combined age-disaggregated risk ratios and published meta-analytic estimates of the case-fatality rate in children who received treatment (19·3%, 95% CI 14·0–26·1)^5^ to produce estimates of tuberculous-meningitis mortality by age group and HIV status. We assumed that untreated tuberculous meningitis was always fatal. We assumed the same relative risk of neurological sequelae among children who had treatment for tuberculous meningitis and lived by age group as for mortality, adjusting to match aggregated meta-analytic estimates to model the age distribution of neurological sequelae (53·9%, 42·6–64·9).^5^

All analyses were done in R version 4.3.1.

### Role of the funding source

The funder of the study had no role in study design, data collection, data analysis, data interpretation, or writing of the report.

## Results

**Figure 3:**
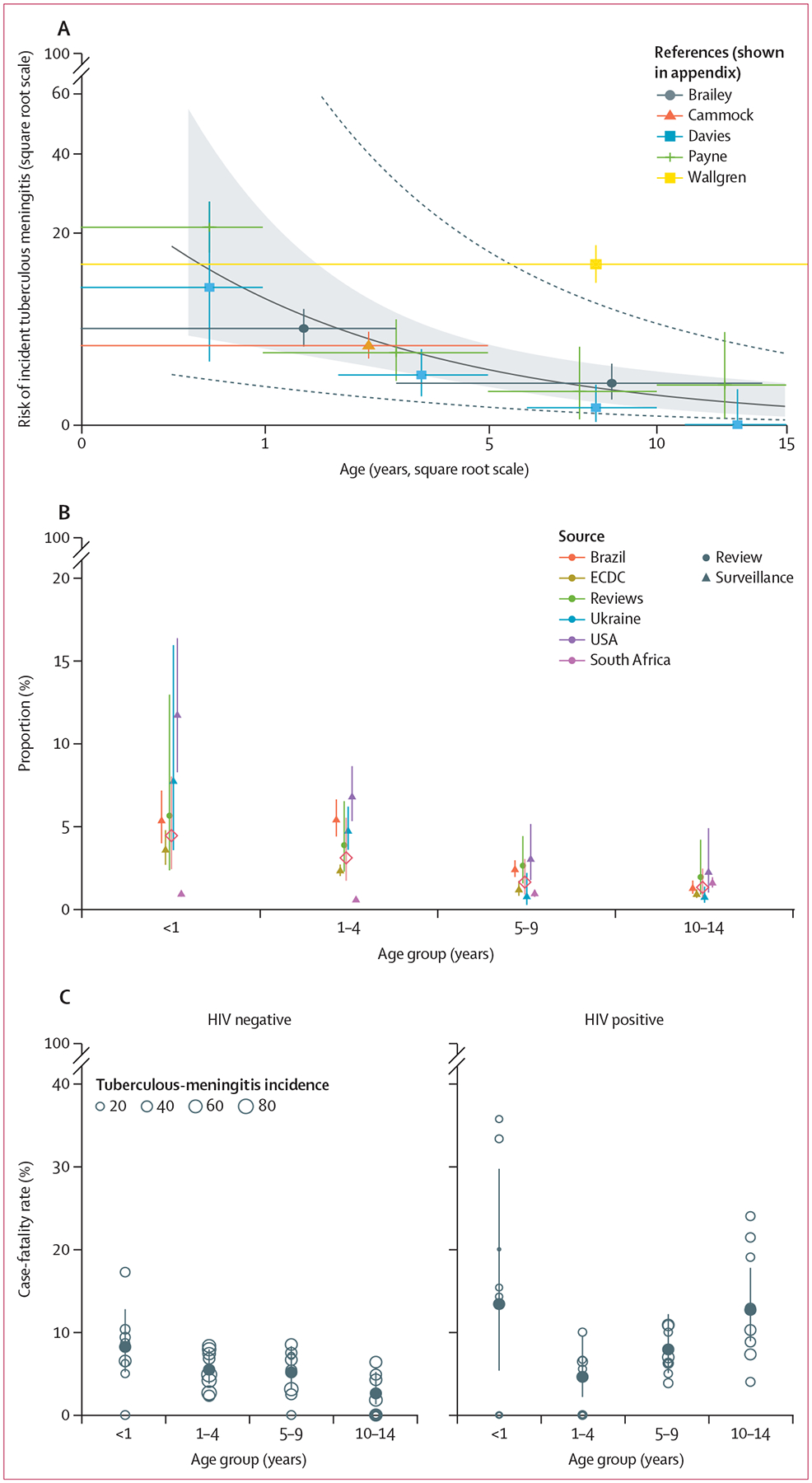
Results of meta-analyses to inform model parameters (A) Risk of incident tuberculous meningitis after *Mycobacterium tuberculosis* infection. Shaded area shows 95% CI of meta-regression. Dashed lines indicate 95% prediction interval. (B) Proportion of tuberculosis that was tuberculous meningitis. Shaded rectangle and diamond show overall results of the meta-analysis. (C) Case-fatality rates by age group and HIV status in children treated for tuberculous meningitis in South Africa, used to estimate age-dependent mortality risk ratios. Open circles show incidence for each year. Solid circles show overall results of the meta-analysis. Lines show 95% CIs. References and data are shown in the appendix (pp 7, 9). ECDC=European Centre for Disease Prevention and Control.

Our literature review of the pre-chemotherapy era identified 15 papers, of which six (40%) reported age-disaggregated data on the risk of progression to tuberculous meningitis after *M tuberculosis* infection (figure 2; appendix p 3). We estimated risk of incident tuberculous meningitis after *M tuberculosis* infection to be highest in children younger than 1 year (figure 3A). We observed a reduction in risk as age increased: 13·6% (95% CI 4·2–31·6) in children younger than 1 year, 3·5% (1·6–6·6) in children aged 1–4 years, 1·0% (0·3–2·1) in children aged 5–9 years, and 0·4% (0·1–1·2) in children aged 10–14 years (appendix p 5). Heterogeneity in the meta-analysis was high (*I*^2^=97·6%).

Our search for published routine surveillance data identified 118 papers. All titles and abstracts were screened and 27 full-text papers were reviewed. We identified seven eligible articles providing published, age-disaggregated tuberculosis and tuberculous-meningitis surveillance data (figure 2; appendix pp 6–7). Our combined dataset of national routine tuberculosis surveillance data included 373 411 children with tuberculosis, of whom 4205 (1·1%) had tuberculous meningitis (figure 2; appendix p 8).

The proportion of tuberculous meningitis in 2019 was highest in children younger than 1 year, slightly lower in children aged 1–4 years, and lowest in children aged 5–14 years (figure 3B; appendix p 9). There were differences between countries with the surveillance data; South Africa had smaller proportions of tuberculous meningitis among tuberculosis notifications in younger age groups than the other included countries. Heterogeneity in the meta-analysis was high (*I*^2^=96·2%).

Our meta-analysis to estimate age-dependent risk ratios for mortality showed that, in children with and without HIV, the case-fatality rate was highest in children younger than 1 year with a gradual decrease with age, although estimates for children with HIV had wide uncertainty (figure 3C). Mortality risk decreased in children aged 1–4 years with HIV but then increased steadily, reaching an almost similar case-fatality rate in children aged 10–14 years as in children younger than 1 year. The largest *I*^2^ for the meta-analyses was 48·5% for children with HIV younger than 1 year (appendix p 11).

An estimated 24 000 (95% credible interval [CrI] 22 300–25 700) children younger than 15 years (78% aged <5 years; 34% aged <1 year) developed tuberculous meningitis in 2019 (table). Of these children, 13 000 (12 100–13 900) were estimated to have been diagnosed and treated for tuberculous meningitis. 9600 (87·3%) of 11 000 untreated children were younger than 5 years. 1724 (7·2%) of the estimated 24 000 children with tuberculous meningitis had HIV.

Among children with tuberculous meningitis, 16 100 (95% CrI 14 900–17 300) were estimated to have died in 2019, a case-fatality rate of 67·0%. Of children who died, 1101 (6·8%) had HIV. 13 380 (83·1%) of 16 100 deaths were estimated to be in children younger than 5 years; 11 000 (68·3%) of 16 100 deaths were estimated to be in children who did not receive tuberculous-meningitis treatment. Of the 7900 (5800–10 000) children who did not die, 5550 (5110–5980) were estimated to have neurological sequelae.

**Figure 4:**
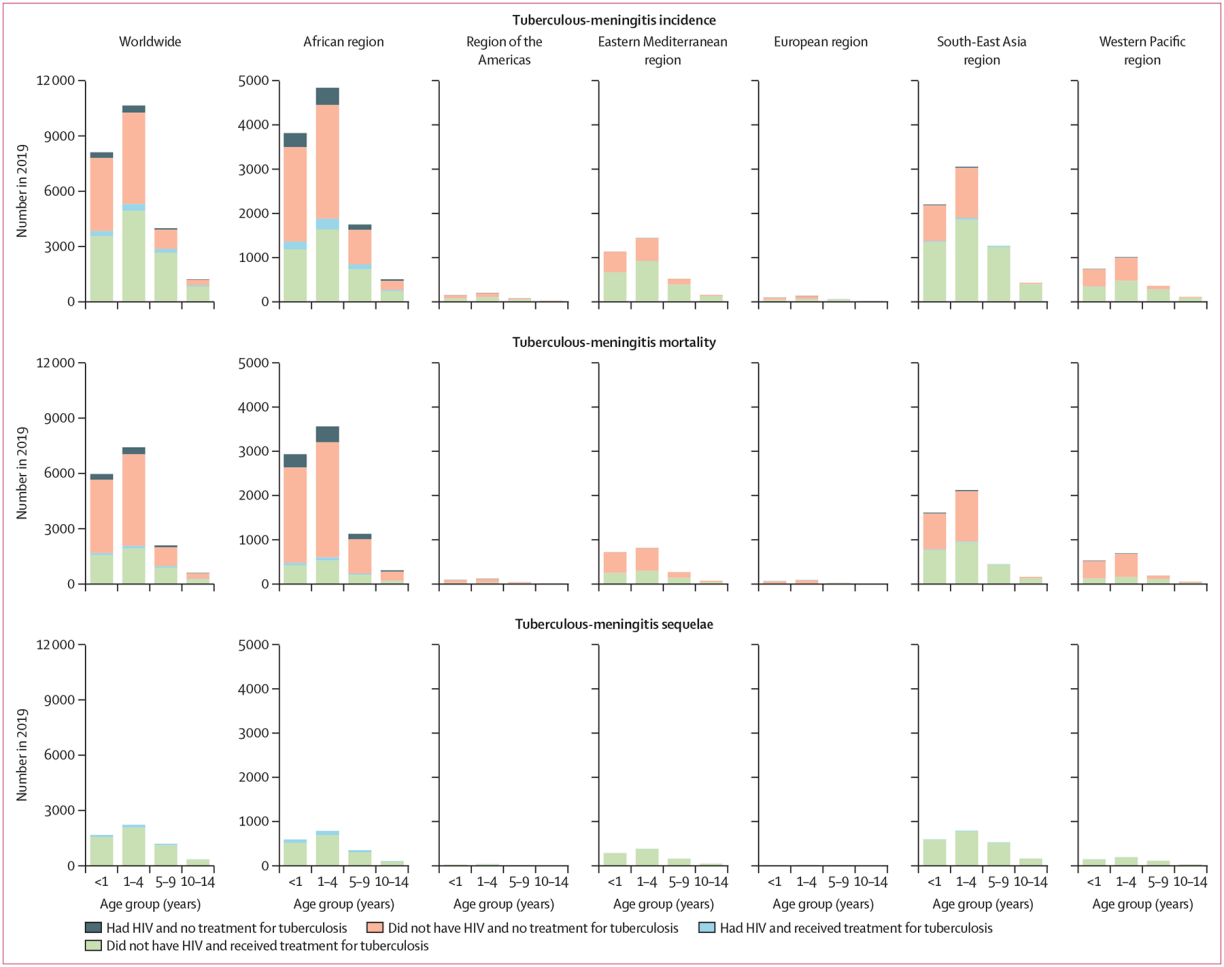
Estimates of tuberculous-meningitis incidence, mortality, and morbidity by age, treatment status, and HIV status in WHO regions in 2019 Please note that scales change between graphs.

The WHO African region (10 900, 95% CrI 10 100–11 700) and South-East Asia region (6930, 5570–8280) contributed the most to incidence of tuberculous meningitis in children in 2019 (African region 10 900 [45·4%] of 24 000 and South-East Asia region 6930 [28·9%]; figure 4). Because of the high proportion of children who were untreated in Africa, the region contributed 7950 (49·4%) of 16 100 global tuberculous-meningitis deaths and had the highest estimated number of deaths (7950, 7290–8600). The African region also had the largest number of incident cases of tuberculous meningitis (1435, 1109–1761) and tuberculous-meningitis deaths (987, 507–1466) among children living with HIV (figure 4; appendix pp 21–22).

## Discussion

This mathematical modelling study provides global and regional estimates of incidence, morbidity, and mortality of tuberculous meningitis in children younger than 15 years in 2019. We estimated that 24 000 children developed tuberculous meningitis in 2019, most of whom were younger than 5 years. Of these children, we estimated that approximately half were diagnosed and treated. We estimated that childhood tuberculous meningitis resulted in 16 100 deaths in 2019 and had a case-fatality rate of 67%, higher than the estimated 19% overall tuberculosis mortality in children in 2019.^17^ Although tuberculous meningitis was a modest absolute proportion of overall incidence (24 000 [2·0%] of the 1 190 000 estimated global burden of tuberculosis in children), it contributed disproportionately to overall tuberculosis mortality (16 100 [7·0%] of 230 000 estimated tuberculosis deaths in children).^17^

In 2019, an estimated 164 000 adolescents and adults aged 15 years or older developed tuberculous meningitis, approximately 1·6% of the global tuberculosis burden in this age group.^18^ 72% of adults received treatment for tuberculous meningitis in 2019, higher than our estimate of 54% of children.^18^ We estimated case-fatality rates to be 67% of children, higher than 48% of adults, and the proportion of people living with HIV was 23% of adults compared with our estimate of 7% of children.^18^

Our study highlights the need for early and improved diagnosis and for improved treatment and prevention to reduce the number of tuberculous-meningitis deaths and tuberculous meningitis-related neurological sequelae in children. We estimated that 11 000 children with tuberculous meningitis died in 2019 because they were not diagnosed or treated, and that almost 90% of those deaths with undiagnosed tuberculous meningitis occurred in children younger than 5 years. By contrast to the typical acute presentation and rapid disease progression of a child with bacterial meningitis, children with tuberculous meningitis often present with a gradual onset of non-specific symptoms, with or without a history of tuberculosis exposure.^19^ A retrospective observational study of 30 children with tuberculous meningitis in South Africa in 2016 found substantial delays in diagnosis, with a median number of four health-care visits before hospital admission despite 70% of children presenting with potential tuberculosis features at the first health-care visit.^20^ Many diagnostic challenges remain for tuberculous meningitis, even at referral centres, as isolation of *M tuberculosis* is elusive and cerebrospinal fluid studies and neuro-imaging, which are crucial in supporting the diagnosis, are often not available in resource-constrained settings.^19^ More resources and training are needed to detect paediatric tuberculous meningitis in high-burden settings and clinicians should have a low threshold to initiate empirical treatment, as studies have shown strong associations between treatment delays and worsened outcomes.^19^

To improve patient outcomes, understanding of optimal treatment for tuberculous meningitis should be improved. Few studies have compared different anti-tuberculosis regimens and doses^19,21^ and there is little evidence on host-directed therapies other than corticosteroids. An improved understanding of the mechanisms underlying tuberculous meningitis-associated inflammation could help identify children at risk of poor outcomes and guide targeted treatment to reduce mortality and morbidity.^22^

Prevention of tuberculosis and tuberculous meningitis rely on a well functioning public health system.^7^ Two preventive strategies for tuberculous meningitis, BCG vaccination and tuberculosis-preventive treatment, are effective yet inconsistently implemented. An analysis of paediatric autopsy data from Ukraine reported undiagnosed, untreated tuberculous meningitis as the cause of death in eight children in a general paediatric hospital, none of whom received BCG vaccination at birth.^23^ BCG vaccination is safe and effective for preventing tuberculous meningitis in children younger than 5 years.^24^ Most tuberculosis-endemic countries recommend BCG vaccination at birth, but implementation has been disrupted by global shortages during 2013–15 and the COVID-19 pandemic, with the pandemic leading to an estimated 33 000 paediatric tuberculosis deaths attributable to not receiving BCG.^25^ Ensuring a steady supply and uptake of BCG is therefore crucial, including during future pandemics.

Tuberculosis-preventive treatment is also a safe and effective strategy to prevent tuberculosis diseases, including tuberculous meningitis, and is particularly integral in children younger than 5 years or who have HIV because of their high risk of disease progression.^26^ A 2020 systematic review and meta-analysis of 137 647 tuberculosis-exposed children showed a 63% effectiveness of tuberculosis-preventive treatment to prevent tuberculosis disease progression.^26^ However, uptake and completion rates of tuberculosis-preventive treatment in settings with high tuberculosis burden remain low. In 2022, only 37% of eligible child contacts younger than 5 years accessed preventive treatment worldwide, resulting in many missed opportunities to prevent both tuberculosis and tuberculous meningitis.^27^ Preventive treatment could not only be cost-effective, but potentially cost saving in some children at high risk of developing the disease, such as infants and children with HIV.^28^ Advances in short-course preventive treatment will make delivery more feasible.^29^

The socioeconomic effects of tuberculous meningitis in a child are substantial for both the family and the health-care system. Children with tuberculous meningitis are often acutely ill at presentation, requiring invasive diagnostic procedures and imaging, admission to hospital for diagnosis, and sometimes intensive care or surgical interventions. Hospitalisation can be for weeks or months in the absence of adequate support to facilitate home-based care. Among children who do not die from tuberculous meningitis, we estimated that 5500 in 2019, most of whom were younger than 5 years, had neurological sequelae that might require lifelong care and support, especially when diagnosed late at advanced stages.^19^ More data on the burden and long-term effects of neurological and behavioural sequelae after tuberculous meningitis on the quality of life of affected families are needed. Caring for a child with tuberculous meningitis also financially affects families by generating substantial health-care costs and impeding caregivers’ ability to work. Future research should include economic evaluations to understand the financial effects of tuberculous meningitis and to provide stronger evidence for dedicated funding and resources for health-care programmes and affected families.

Our findings have important implications for programmes in settings with high tuberculosis burden. Strengthening routine reporting and surveillance data for tuberculous meningitis by including tuberculous meningitis with staging information as a reporting indicator will allow for quantification of notified cases in children, adolescents, and adults. Routine surveillance data on tuberculous meningitis can assist national tuberculosis programmes with monitoring and evaluation and overall progress in tuberculosis control, as well as guide planning and budget allocation to ensure that the health-care needs of children who do not die from tuberculous meningitis are met.^7^

Our study has several limitations. First, we were unable to find studies published since 1980 that identified children exposed to tuberculosis and followed them up without preventive treatment to an endpoint of tuberculous meningitis. As a result, we were limited to pre-chemotherapy literature (ie, before 1950) to inform the model parameter on the risk of tuberculous-meningitis progression after *M tuberculosis* infection. Second, the methods of the included studies were not always consistently and clearly defined and reported, and publications from the early and mid 1900s cannot always be searched systematically. Third, the populations from pre-chemotherapy literature might not generalise to current populations due to the absence of HIV infection and BCG vaccination, so we included them as additional parameters in our model. Fourth, we did not include other potential risk factors, such as nutrition, due to the paucity of available data. Fifth, the routine surveillance data we used were from settings that might not be representative. However, to mitigate this limitation, we used multiple approaches in our model and used priors for variation in tuberculosis incidence and case detection as a proxy for the functionality of health-care systems. Sixth, statistical heterogeneity in some of our meta-analyses were high. Seventh, the South African dataset was the only dataset that included mortality and HIV status by age. These data were therefore the only data that could inform relative mortality and morbidity between the different age groups, not the outcomes themselves. However, in the meta-analysis of the proportion of tuberculosis that was tuberculous meningitis overall, South African data were substantially different from other data, especially for younger age categories. The reasons for this discrepancy are unclear, but could be related to different reporting practices across provinces. Eighth, we did not include estimates of tuberculous meningitis in adolescents aged 15–19 years. They are, however, a priority group, at high risk of tuberculosis and HIV infection, and should be included in future work. Ninth, due to scarce available data to inform estimates of diagnostic accuracy for tuberculous meningitis in different settings, we had to rely on the estimates of tuberculosis case detection in children for all tuberculosis. However, we only used these estimates to define a probabilistic relationship between notifications and incidence to account for variability in diagnostic services, and we acknowledge the substantial uncertainty around incidence estimates. Tenth, in the absence of any data, we had to assume notification was synonymous with detection and treatment, but we know that this is not completely accurate because of the limitations in surveillance systems. For example, children diagnosed with tuberculous meningitis in hospitals might be more likely to be excluded from routine surveillance data.^30^ This assumption would have resulted in our incidence estimates being lower than the true burden of tuberculous meningitis, but could have led to overestimating deaths if more children with tuberculous meningitis were treated but not reported.

Estimates of how many children and families are affected by tuberculous meningitis globally provide important data to advocate for this vulnerable subgroup of children with tuberculosis. Our findings highlight the need for sensitive and specific diagnostic tools and strong health-care systems to facilitate early diagnosis, as many of these children were estimated to have died without a diagnosis. They also support tuberculosis-prevention strategies to be considered as a public health priority, especially in young children and children with HIV. As most children who developed tuberculous meningitis were young and had neurological sequelae if they did not die, future research should measure the long-term economic and societal costs of tuberculous meningitis and the effects on the quality of life of the children and their families.

## Supplementary Material

1

## Figures and Tables

**Table:** Global estimates of annual incidence, mortality, and neurological sequelae of tuberculous meningitis in children in 2019, by age group

	Younger than 1 year	Aged 1–4 years	Aged 5–9 years	Aged 10–14 years	Aged 0–14 years
**Estimated annual incidence**					
Treated children	3850 (3350–4350)	5330 (4770–5880)	2880 (2440–3320)	910 (663–1160)	13 000 (12 100–13 900)
Untreated children	4270 (3100–5430)	5330 (4060–6600)	1120 (420–1820)	320 (0–723)	11 000 (9130–12 900)
Total	8120 (7060–9170)	10 700 (9510–11 800)	4000 (3450–4540)	1230 (913–1550)	24 000 (22 300–25 700)
**Estimated annual mortality**					
Treated children	1700 (280–3120)	2080 (555–3610)	992 (248–1740)	305 (0–732)	5080 (2820–7340)
Untreated children	4270 (3100–5430)	5330 (4060–6600)	1120 (420–1820)	320 (0–723)	11 000 (9130–12 900)
Total	5970 (5160–6780)	7410 (6570–8260)	2110 (1850–2370)	625 (483–767)	16 100 (14 900–17 300)
**Estimated annual morbidity**					
Neurological sequelae among treated children who did not die from tuberculous meningitis	1700 (1460–1940)	2250 (1970–2520)	1220 (1010–1430)	381 (273–489)	5550 (5110–5980)

Data are n (95% credible interval).

## Data Availability

Code and all data to reproduce this study, except raw country-level surveillance data, are available at https://github.com/petedodd/TBMK.

## Research in context

### Evidence before this study

Childhood tuberculous meningitis has a high case-fatality rate and substantial morbidity, yet data are scarce regarding how many of the estimated 1·25 million children with tuberculosis each year develop tuberculous meningitis worldwide. We searched PubMed from database inception to Aug 5, 2024, for abstracts published in English using the search terms (“tuberc*”[Title/Abstract]) AND (“meningitis”[Title/Abstract]) AND (“incidence”[Title/Abstract] OR “burden”[Title/Abstract]) AND (“model*”[Title/Abstract] OR “estimate*”[Title/Abstract]). Only modelling studies that reported the burden of tuberculous meningitis in children were included. Of 138 articles identified, only one modelling study estimated the burden of tuberculous meningitis in adults and no studies quantified the global burden of tuberculous meningitis in children. A systematic review and cost-effectiveness analysis found that BCG vaccination prevented approximately 30 000 cases of tuberculous meningitis during the first 5 years of life in children born in 2002; however, global or regional estimates of tuberculous meningitis were not provided. Moreover, study-level data in this meta-analysis included effect estimates for BCG vaccination but not for several other necessary variables, including risk of tuberculous meningitis after primary *Mycobacterium tuberculosis* infection, proportion of notified childhood tuberculosis that was tuberculous meningitis, or increased risk of death due to tuberculous meningitis.

### Added value of this study

To our knowledge, ours is the first study to estimate global data for tuberculous meningitis and its outcomes in children younger than 15 years. We estimated that 24 000 children developed tuberculous meningitis in 2019 but 11 000 did not receive treatment. We also estimated that 16 100 children with tuberculous meningitis died and 5550 children had neurological sequelae. Most children were younger than 5 years, with 18 820 incident cases of tuberculous meningitis and 13 380 deaths in this age group.

### Implications of all the available evidence

Outcomes are improved when tuberculous meningitis is diagnosed and treated early. In settings with a high tuberculosis burden, clinicians should be alert for tuberculous meningitis in unwell young children, including determining history of tuberculosis exposure, to facilitate early diagnosis. Tuberculous meningitis is not currently routinely differentiated from tuberculosis in notification data (ie, routine surveillance data that countries report to WHO). Reporting these data and having staging information would be valuable for monitoring and evaluation as they indicate how well health-care systems are functioning. Increased coverage of available prevention strategies, including BCG vaccination at birth and tuberculosis-preventive treatment after exposure, are crucial as we should use every possible opportunity to prevent this disease. Further research to develop improved diagnostic tools for tuberculous meningitis and more effective management strategies to reduce the high mortality and morbidity of tuberculous meningitis are needed.
