# Supplementary material for: Global burden of tuberculous meningitis in children aged 0–14 years in 2019: a mathematical modelling study
Source: Lancet Glob Health. Author manuscript; Available in PMC 2025 Jan 14. (PMC11729397; doi:10.1016/S2214-109X(24)00383-8)
Supplement: 1 [file NIHMS2043426-supplement-1.pdf]

THE LANCET
Global Health

Supplementary appendix

This appendix formed part of the original submission and has been peer reviewed. We post it as supplied by the authors.

Supplement to: du Preez K, Jenkins HE, Martinez L, et al. Global burden of tuberculous meningitis in children aged 0–14 years in 2019: a mathematical modelling study. *Lancet Glob Health* 2025; **13:** e59–68.

The global burden of tuberculous meningitis in children aged 0-14 years: a mathematical modelling study

Appendix and supplementary results

Table of Contents

Overview 2

Risk of incident TBM following *M.tb* infection 2

Meta-analysis to determine the risk of TBM disease progression following infection 4

TBM as a proportion of tuberculosis notifications in children 5

Literature search for published tuberculosis and TBM surveillance data 5

National tuberculosis and TBM surveillance data 8

Meta-analysis of the proportion of notified tuberculosis that is TBM 8

Meta-analysis of eligible, published tuberculosis surveillance data (Step 1a) 8

Meta-analysis of national tuberculosis and TBM surveillance data (Step 1b) 9

Meta-analysis combining national and published surveillance data (Step 2) 10

The influence of HIV 11

Case fatality ratios 11

The influence of HIV 13

Notification splits 14

Notification age-split 14

Notification HIV splits 15

Mathematical modelling of TBM incidence, morbidity, and mortality 16

Other input data sources & preparation 16

Modelling approach 16

Code and data availability 19

Acknowledgements 19

Supplementary results 20

References 23

Overview

Tuberculous meningitis (TBM) is fatal if untreated and causes life-long disability in survivors. Limited surveillance data are available, and there are no estimates of the global burden of TBM in children (<15 years). We reviewed the literature and sourced routine notification data to quantify: 1) children's risk of progression to TBM following Mycobacterium tuberculosis (*M.tb*) infection, 2) TBM as proportion of notified childhood tuberculosis and 3) risk ratios for TBM mortality by age. We used these as priors in a Bayesian model to estimate TBM incidence at the country-, regional-, and global-level in 2019. The model included *M.tb* infection, progression to disease, detection and outcomes by age, tuberculosis-treatment and HIV-infection status. In addition, we estimated the total number of children with TBM that either died or survived with neurological sequalae.

Risk of incident TBM following *M.tb* infection

Natural history studies from the pre-chemotherapy era quantified the risk of TBM following *M.tb* infection. We used a review of the pre-chemotherapy literature by Marais et al[1] to identify possible studies. The review included original studies on childhood tuberculosis conducted between 1920 and 1950, focussed on those published in English, had at least a 10-year study duration and reported on more than 1,000 children. Three exceptions were made to ensure wide representation of the spectrum of disease – these are described in detail in the original article.[1] The review however only reported combined estimates of age-related risk for disease progression to both TBM and/or miliary tuberculosis. We therefore sourced and reviewed the original papers included in the Marais review, as well as references listed in these papers, to identify studies that followed a defined number of children with evidence of recent *M.tb* infection and that reported on TBM distinctly by age. We excluded papers that did not report TBM separately to miliary tuberculosis.

In order to estimate the risk of progression to TBM following TB infection, it is necessary to use data from studies that determined TB infection status and then followed children carefully to determine the incidence of TBM, unimpacted by TB therapy. Therefore, the studies we used from the pre-chemotherapy era provide the only data source available. Since the 1970s TB preventive treatment (TPT) has been shown to be safe and effective in preventing TB disease progression following TB exposure or infection, and therefore any contemporary studies that identified children with TB infection would have ethically had to provide TPT to these children which would alter the risk of disease progression. While much of the natural history data is from the US and the UK, at the time these studies were conducted, the TB rates and annual risk of TB infection in the US and UK were very similar to what is observed today in many high TB burden countries. For this reason, we used multiple approaches to triangulate the risk and to take into account the variation in TB incidence and the functionality of healthcare systems.

We identified a total of 15 papers that could potentially contribute data. Three of the authors (KDP, HJ and LM) reviewed all 15 papers and decided jointly whether the data reports the risk of TBM disease progression by age following infection. We identified 6 papers that reported TBM distinctly by age – 3 of the 9 papers included in the original review[2-4] and a further 3 from the references[5-7]. One of the identified studies, Miller, et.al 1960[7], reported on a sub-set of data included in the paper by Cammock and Miller, 1953[5], and was therefore not included. Data was extracted by two of the three authors separately, and any inconsistencies were resolved by joint discussion. Table A1 provides an overview of the 5 studies that were included, as well as the data that they contributed.

Table A1. Overview of studies included in the meta-analysis of the risk of TBM disease progression following infection[1]

	Study description	Study setting and period	Age groups	Number of children infected	Number of TBM cases
Wallgren 1938[2]	The paper reports on 453 infants and children 0-16 years who had been under observation and treatment from the onset of primary tuberculosis.	Study setting and period not specified	0 - 16	453	64
Brailey 1943[3]	Included children <15 years of age with evidence of tuberculosis infection/disease; one of the children in a family had to be younger than 2 years	Baltimore, USA<br>1928-1937	0 - <3<br>3 - 14	512<br>636	26<br>6
Davies 1961[4]	Contacts of tuberculosis cases attending the "Child Contact Clinic" at Brompton Hospital and Chelsea Hospital (latter is small). Excluded any found to have lesions before visit to clinic but included if has lesions *when* they visited.	Brompton and Chelsea Hospitals, London, UK<br>Jan 1930 – Dec 1952	0-1 years<br>2-5 years<br>6-10 years<br>11-15 years	29<br>369<br>634<br>535	3<br>5<br>1<br>0
Cammock and Miller 1953[5]	The study included all children born in Newcastle between 1 Jan 1945 - Dec 31, 1949. Details from all hospitals, chest and contact clinics to the end of Dec 1951 identified 258 children (2-year observation period). Their illness was placed against the number of children in the population who had acquired primary infection in the 5 years. The Department of Child health completed tuberculin surveys and documented conversions in Newcastle children 1947-52.	New Castle, England<br>1945-1951	0 - <5	1020	35
Payne 1959[6]	Included children with a known time of tuberculosis infection who had been infected at least two years previously. Information was recorded about health and supervision from the time of infection. At the beginning of the study all children had radiological examination of the chest, abdomen, and neck and, if necessary, of any other region.	Tyneside, UK<br>1956-1957	0 - <1<br>1 - <5<br>5 - < 10<br>10 - <15	229<br>211<br>165<br>116	49<br>6<br>1<br>1

[1]*At the time these studies were conducted in the US and the UK, the TB rates and annual risk of TB infection in the US and UK were very similar to what is observed today in high TB burden countries.*

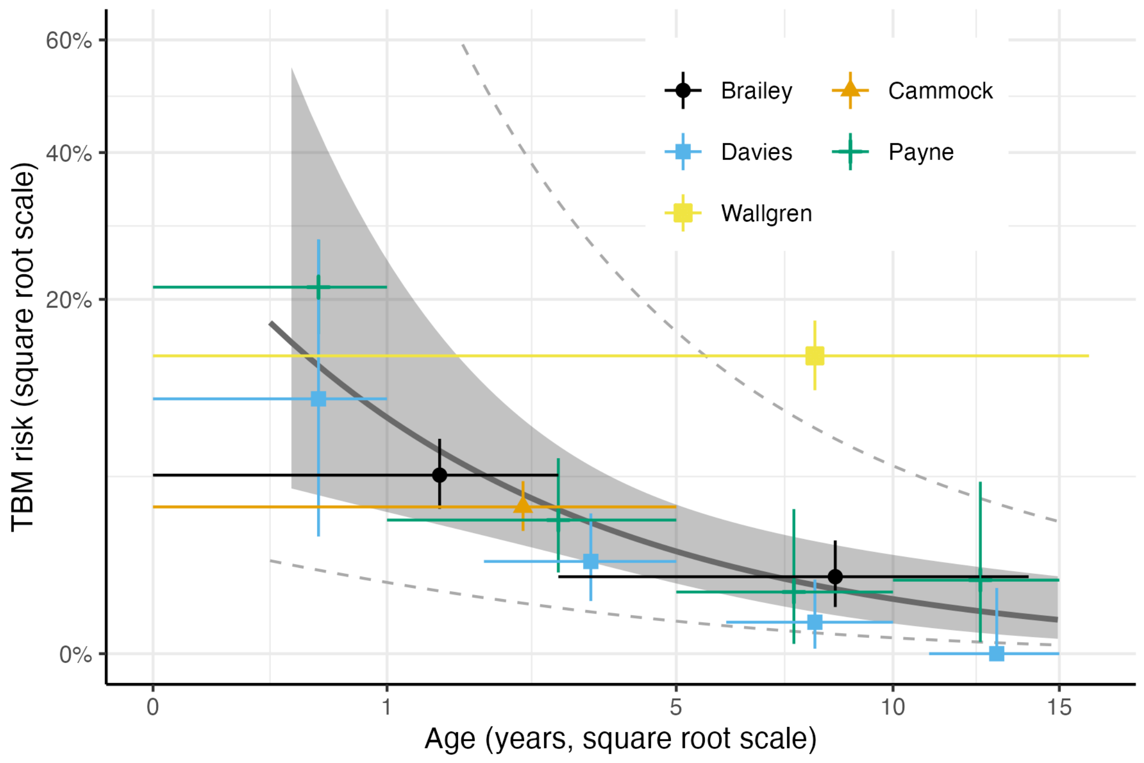

Figure A1 Meta-analysis of progression risk to TBM[2-6]

Because of the different age categories used in these studies, we used a continuous model for the dependence on age, by examining empirical plots, we found the square root of age resulted in a better fit than using age as an explanatory variable (see Figure A1**)**. We thus used an underlying continuous model of age response to manage the disparate age categories and produce predictions for standardized age groups, using the assumption that response for a given age group was the uniform average of the underlying continuous response across the group, i.e. we modelled the probability of progression p for an age category from A_1 to A_2 by

$$logit(p) = \frac{1}{A_2 - A_1}\int_{A_1}^{A_2} f(a)\, da$$

right hand side was computed analytically, and the coefficients (intercept and slope for square root-age for *f(a)* – a form suggested by visual comparisons) inferred from a binomial random effects model. We then used the same approach to predict response for the standard age categories we use in our model. As Wallgren reported only on the full age range, and resulted in an outlier, we repeated this analysis excluding Wallgren as a sensitivity analysis (see Table A2). Heterogeneity was high (I^2=97.64%).

Table A2 Estimated TBM progression risks, with and without Wallgren.
SD=standard deviation; CI=confidence interval

Analysis	Age category (years)	Progression risk	SD of progression risk	Lower 95% CI	Upper 95% CI
With Wallgren	<1	13.55%	7.23%	4.16%	31.63%
	1-4	3.53%	1.29%	1.64%	6.56%
	5-9	0.96%	0.48%	0.34%	2.12%
	10-14	0.38%	0.32%	0.07%	1.24%
Without Wallgren	<1	14.41%	2.92%	9.52%	20.79%
	1-4	2.77%	0.43%	2.01%	3.70%
	5-9	0.50%	0.16%	0.26%	0.89%
	10-14	0.14%	0.07%	0.05%	0.31%

TBM as a proportion of tuberculosis notifications in children

Literature search for published tuberculosis and TBM surveillance data

We wanted to identify any studies that reported routine tuberculosis surveillance data in children and included the number of children with TBM. We searched PubMed on 7 July 2022 using the following search terms: ("childhood tuberculosis"[Title/Abstract] OR "pediatric tuberculosis"[Title/Abstract] OR "paediatric tuberculosis"[Title/Abstract] OR ("tuberculosis"[Title/Abstract] AND "child*"[Title/Abstract])) AND ("meningitis tuberculosis"[Title/Abstract] OR "tuberculosis meningitis"[Title/Abstract] OR "tuberculous meningitis"[Title/Abstract] OR “extrapulmonary”[Title/abstract]) AND ("epidemiology"[Title/Abstract] OR "surveillance"[Title/Abstract])

We included studies that were published in English and reported: 1) national data or data from a well-defined geographical area; 2) data from the year 2000 onwards, 3) the number of children with tuberculosis and the number with TBM, and 4) data in at least two age categories, 0-4 and 5-14 years. Abstracts of all articles were screened, and were included for full text review if the above inclusion criteria was not clearly violated from the abstract. If paediatric data was included in the study, and if any spectrum of disease (including CNS-tuberculosis or extrapulmonary tuberculosis) was reported in the abstract, the full text was reviewed. TBM did not specifically have to mentioned in the abstract to be eligible for full text review.

The PubMed keyword search using titles and abstracts identified 116 possible papers. Two additional, relevant studies, not identified by this search, but known to the authors were also included.[11,13] A total of 118 abstracts were screened by one author (KDP) to identify eligibility, and 27 full-text papers were reviewed. Seven articles met the inclusion criteria. Figure 1 provides a flow diagram and reasons for exclusion. Data were extracted from all 7 articles and included in the meta-analysis. Detailed information of the eligible and included studies are provided in Table A3. Heterogeneity was high (I^2=96.20%).

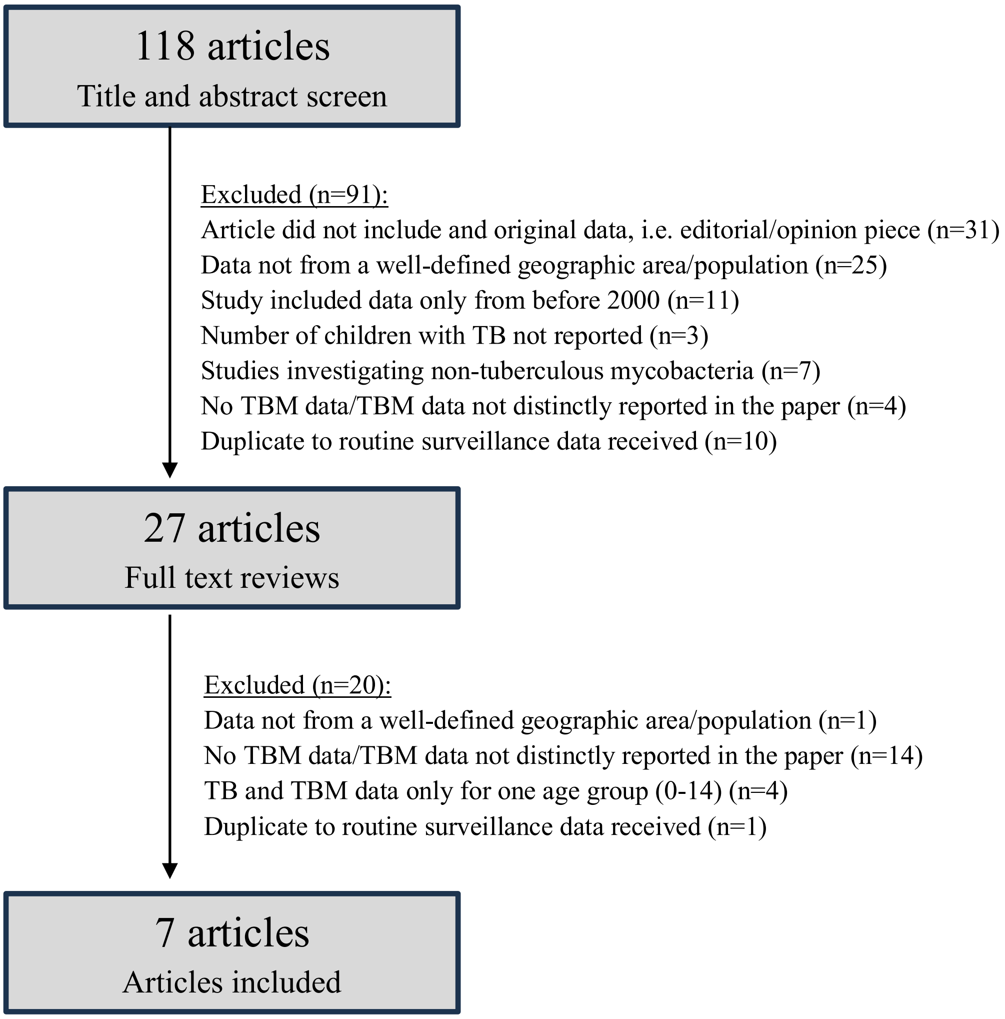

Figure A2. Flow diagram of literature search results and reasons for exclusion

Table A3. Overview of published studies included in the meta-analysis to determine the proportion of childhood tuberculosis that is TBM

	Study population and period	Study description	Age groups	Number of children with tuberculosis	Number of children with TBM
Chu P, et.al.[8]	China<br>Oct 2015 - Dec 2018	Discharge data from the Futang Research Centre of Pediatric Development – a multi-tiered pediatric diagnosis and treatment network and affiliated with the National Centre for Children's Health, China, of all paediatric tuberculosis patients from 22 hospitals (9 provincial and 13 municipal) across all six regions in China	<1<br>1-3<br>4-6<br>7-<18	527<br>1 748<br>793<br>1 585	58<br>328<br>139<br>203
Donnan EJ, et.al.[9]	Queensland, Australia<br>2005-2014	Data collected in the Queensland Notifiable Conditions System	0-<2<br>2-<5<br>5-<10<br>10 - 16	17<br>22<br>29<br>59	2<br>1<br>1<br>3
Ducomble T, et.al.[10]	Germany<br>2002-2009	National tuberculosis surveillance data	0-4<br>5-9<br>10-14	882<br>460<br>388	34<br>10<br>5
Erkens CGM, et.al.[11]	Netherlands<br>2005-2012	Routinely notified tuberculosis cases from the Netherlands tuberculosis register	<5<br>5-14	154<br>249	3<br>4
Mohiyuddin T, et.al.[12]	United Kingdom<br>2000-2015	All tuberculosis cases notified to the enhanced tuberculosis surveillance system (ETS) in England, Wales and Northern Ireland and the enhanced surveillance of mycobacterial infections in Scotland.	<5<br>5-<15	2 177<br>4 035	85<br>79
Onyango DO, et.al.[13]	Kenya<br>2013-2015	National tuberculosis program data from Kenya	0-4<br>5-9<br>10-14	11 160<br>5 799<br>6 788	101<br>59<br>72
Wang PD[14]	Taipei City, Taiwan<br>1998-2005	National tuberculosis Registry Database	0-4<br>5-9<br>10-14	48<br>39<br>76	1<br>0<br>1

National tuberculosis and TBM surveillance data

We obtained surveillance data with appropriate age stratification for four countries (Brazil=BRA, United States of America=USA, Ukraine=UKR, South Africa=ZAF) and in addition from the European Centre for Disease Prevention and Control (ECDC):

Table A4. National tuberculosis and TBM surveillance datasets included in the meta-analysis

Source	Years	TBM cases	Tuberculosis cases
BRA	2001 - 2019	653	20719
USA	2014 - 2018	116	2085
UKR	2015 - 2020	93	3719
ZAF	2009 - 2016	2884	320127
ECDC	2010 - 2019	459	26761

For the ECDC data, we took TBM to be defined as tuberculosis with either major or minor site of tuberculosis recorded as meningitis. We excluded records with sex unknown and HIV status know to be positive, some countries had zero TBM cases in the data, and following discussion with data providers, we excluded these as countries that had not reported on TBM. This left 29 countries contributing (Iceland, Malta, Luxembourg, Cyprus, Estonia, Slovenia, Croatia, Czechia, Hungary, Ireland, Norway, Denmark, Switzerland, Greece, Austria, Latvia, Slovakia, Sweden, Netherlands, Lithuania, Portugal, Belgium, Poland, Bulgaria, Germany, Italy, France, Spain, Romania), which we aggregated to consider as a single country.

All data were aggregated into common age-groups: <1 year, 1-4 years, 5-9 years, 10-14 years. South Africa and Brazil had stratification by HIV for use in analysis for children living with HIV. Elsewhere, children living with HIV were excluded from the data if possible, or else assumed to be a negligible contribution to the data.

Meta-analysis of the proportion of notified tuberculosis that is TBM

We used a two-step approach to generate meta-analytic estimates of the proportion of tuberculosis notifications that are TBM.

1. Meta-analysis for published surveillance data or for national/country surveillance data:
    a. For published data: a random effects meta-analysis across studies
    b. For country data: random effects binomial meta-analysis over years, with data restricted to HIV-negative and aggregated to our age groups
2. Random effects analysis of estimates from step 1. That is, the point estimates and standard errors from step 1 for each category are considered as input data for a second random-effects meta-analysis (step 2), fitted with restricted maximum likelihood to give combined final results.

All meta-analyses were done in R with the metafor package (version 4.0.0).

Meta-analysis of eligible, published tuberculosis surveillance data (Step 1a)

Data were not stratified simultaneously by age and year, and various age categories were reported. As with the meta-analysis for risk of progression, we used a continuous response proportional to the square root of age, with mean response in individual studies depending on the mean of this continuous age

response over the stated age categories (see Figure A3). Predictions were generated for our standard age categories for use in the second state meta-analysis.

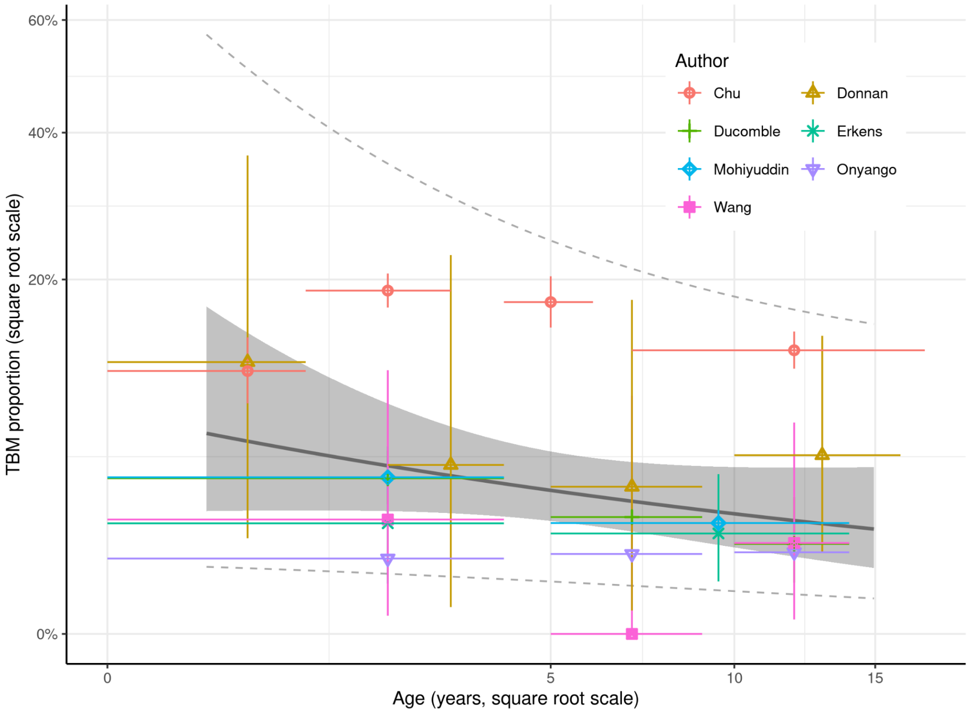

Figure A3 Meta-analysis of the proportion of tuberculosis notifications that are TBM from published data/literature review[8-14]

The horizontal bars represent age categories used by each study, and the vertical bars the 95% confidence interval of each study's estimate

Meta-analysis of national tuberculosis and TBM surveillance data (Step 1b)

Meta-analytic estimates of the proportion of tuberculosis notifications that are TBM were computed with a random effect for year (see Figure A4) as Step 1b of our two-step meta-analysis.

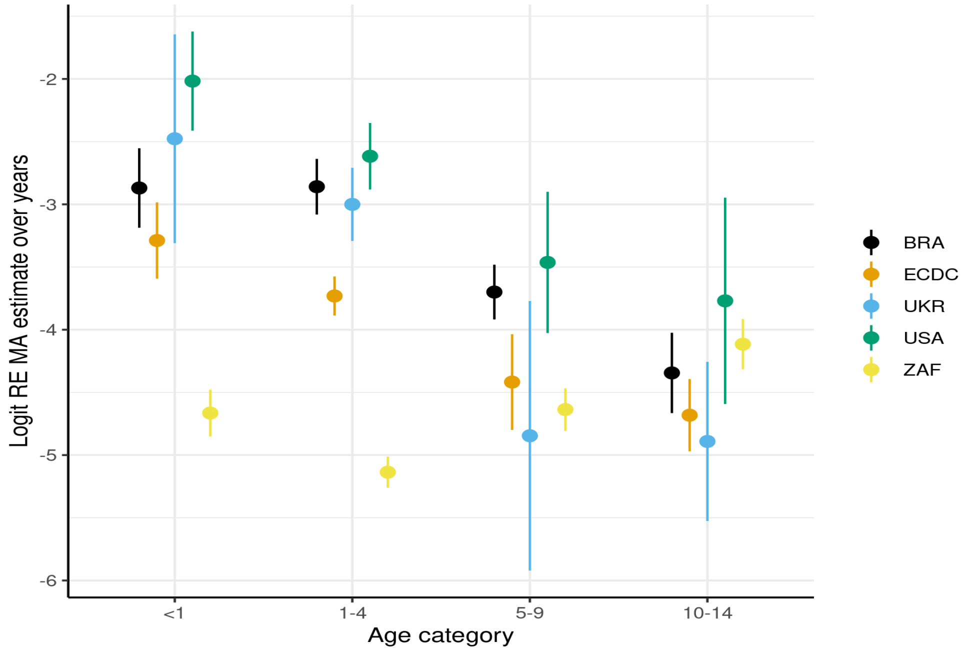

Figure A4 Meta-analysis of individual national surveillance data

RE=random effects; MA=meta-analysis

Meta-analysis combining national and published surveillance data (Step 2)

The meta-analytic estimates of the proportion of tuberculosis notifications that is TBM in each age category were combined with a second random-effects meta-analysis, that treated the logit proportion for each country as an input on an equal par with the ECDC estimate, and the literature estimate. For step 2, a random effects meta-analysis was fitted on a logit scale with restricted maximum likelihood, using the step 1 pooled estimates and standard errors as inputs. The combined estimates are shown in see Figure A5 and Table A5.

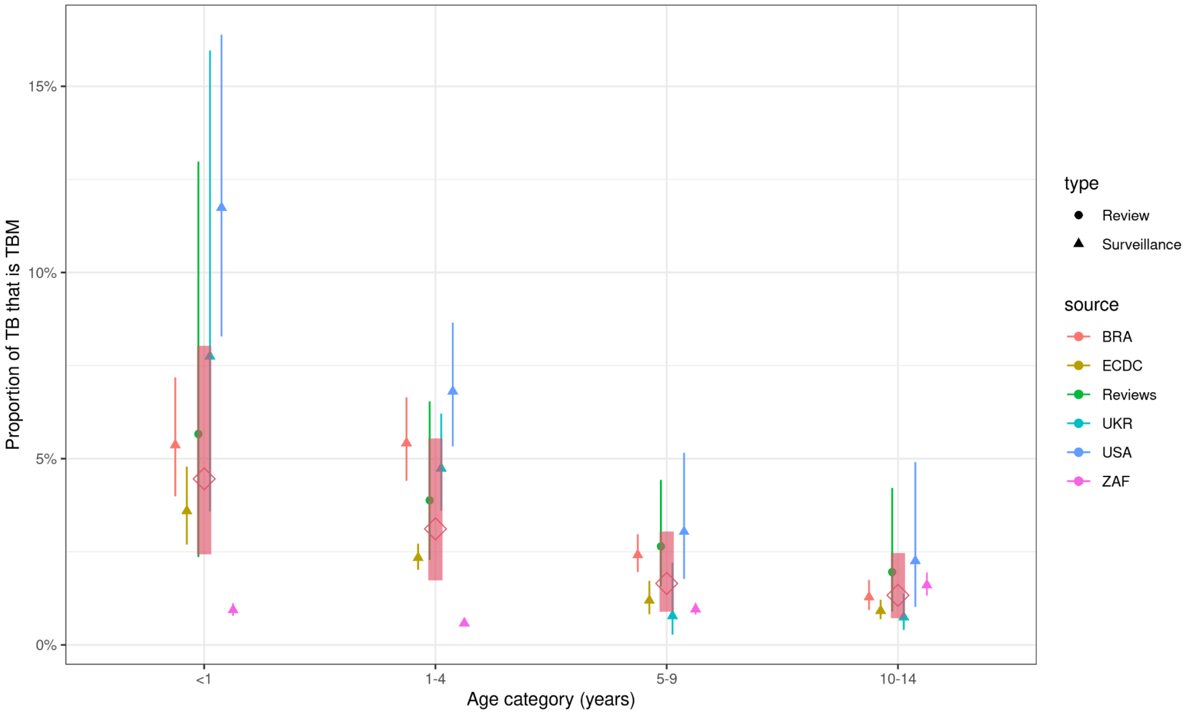

Figure A5 Meta-analysis combining individual country surveillance data and review data. Red diamond and ribbon represent overall meta-analysis results and uncertainty.

Table A5 Meta-analytic estimates of the proportion of notified tuberculosis that is TBM

Age category (years)	Proportion	Lower 95% CI of proportion	Upper 95% CI of proportion
<1	4.46%	2.43%	8.03%
1-4	3.12%	1.73%	5.54%
5-9	1.65%	0.89%	3.05%
10-14	1.33%	0.71%	2.47%

The influence of HIV

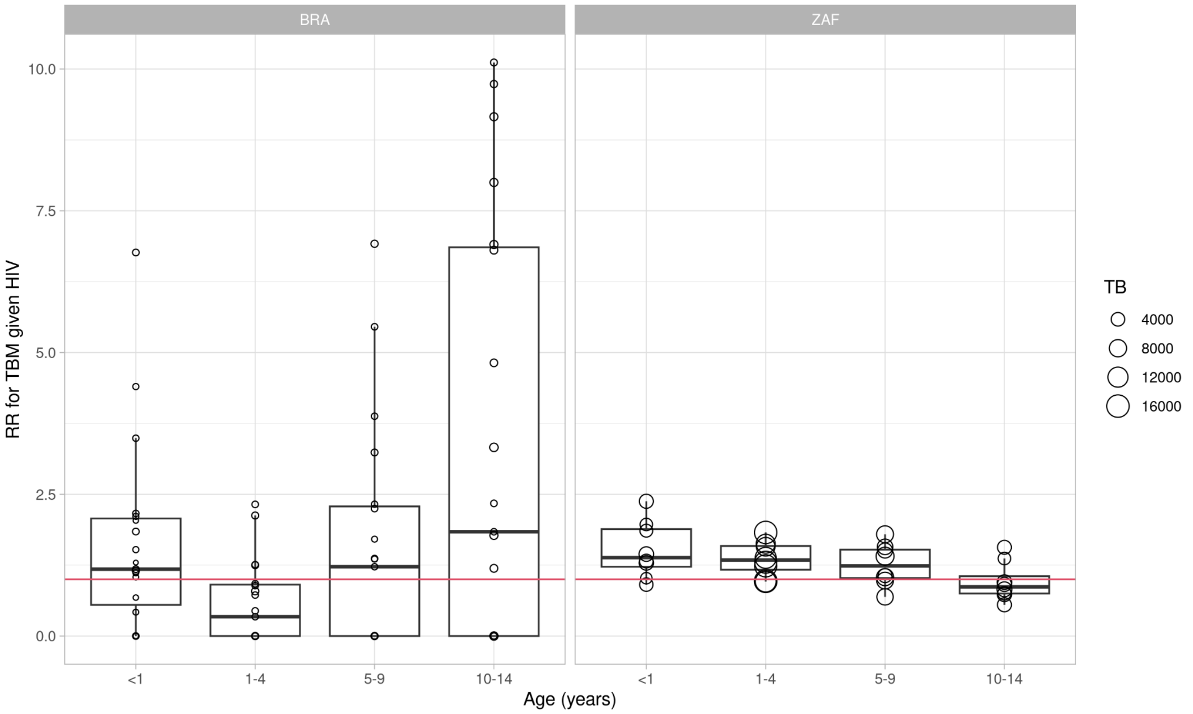

Figure A6 Empirical risk ratios for tuberculosis notifications being TBM given HIV in each year

We performed a random effects meta-analysis of the South African data (Figure A6) to produce an age-independent odds ratio of tuberculosis notifications being TBM given HIV: 1.37 (95%CI 1.22 to 1.55). Numbers were judged too low to for the Brazil data to attempt a synthesis.

Case fatality ratios

We used South African data to estimate the age-specific relative probability of death (see Figure A7). Because the overall estimated case fatality ratio (CFR) for South Africa differs systematically from that found in systematic review and meta-analysis for reasons discussed in the main article, we used the age-specific data in South Africa to produce a random effects meta-analytic estimate of the odds ratio of death (relative to Chiang et al's[15] summary estimate): see Table A6. We also applied these odds ratios to the meta-analytic risk of TBM sequelae in Chiang et al[15] to obtain age-specific estimates. The associated I2 stats were 48% for CLHIV <1 year if age, 3.8% for CLHIV 10-14 years of age, and zero up to within numerical accuracy for all other categories.

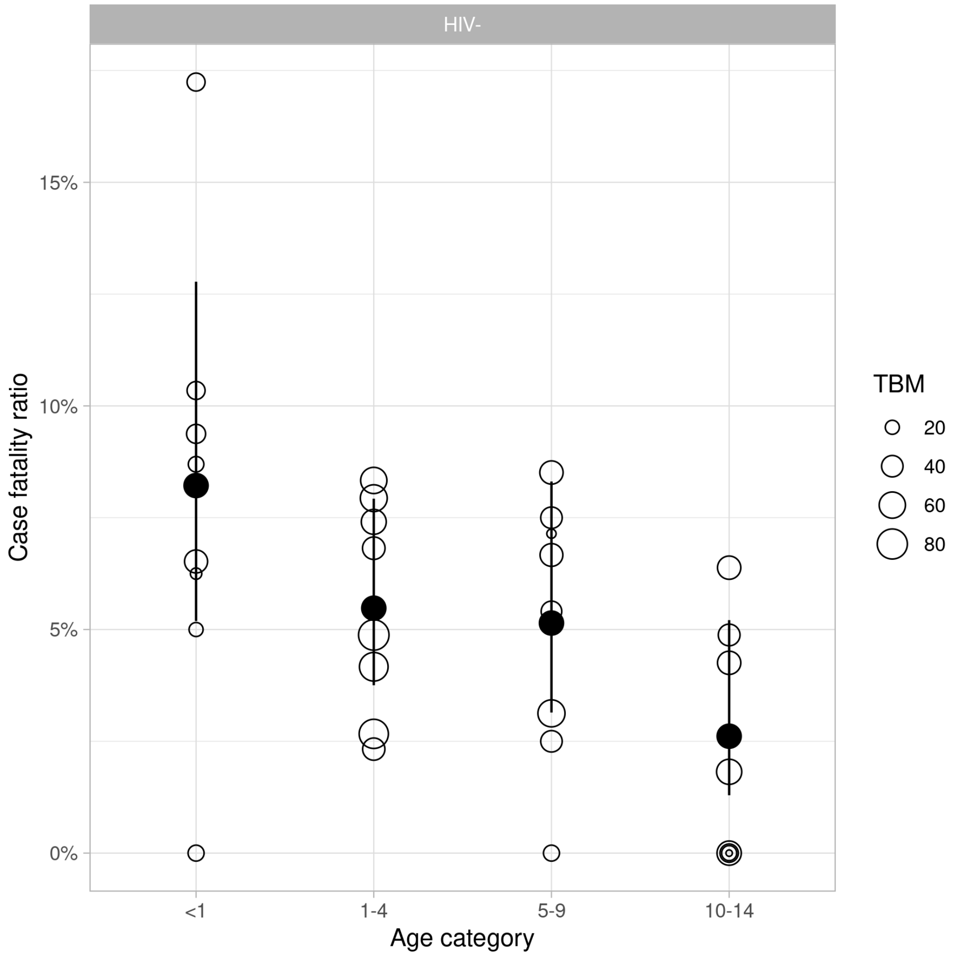

Figure A7 Case fatality ratios as the ratio of TBM deaths to notification data in South Africa
Note: Solid black circles represent pooled estimates.

Table A6 Meta-analytic odds ratios of age-specific case fatality ratios

Age category (years)	OR	Lower 95% CI for OR	Upper 95% CI for OR
**<1**	1.64	1.01	2.65
**1-4**	1.06	0.72	1.56
**5-9**	0.99	0.60	1.64
**10-14**	0.49	0.24	0.99

The influence of HIV

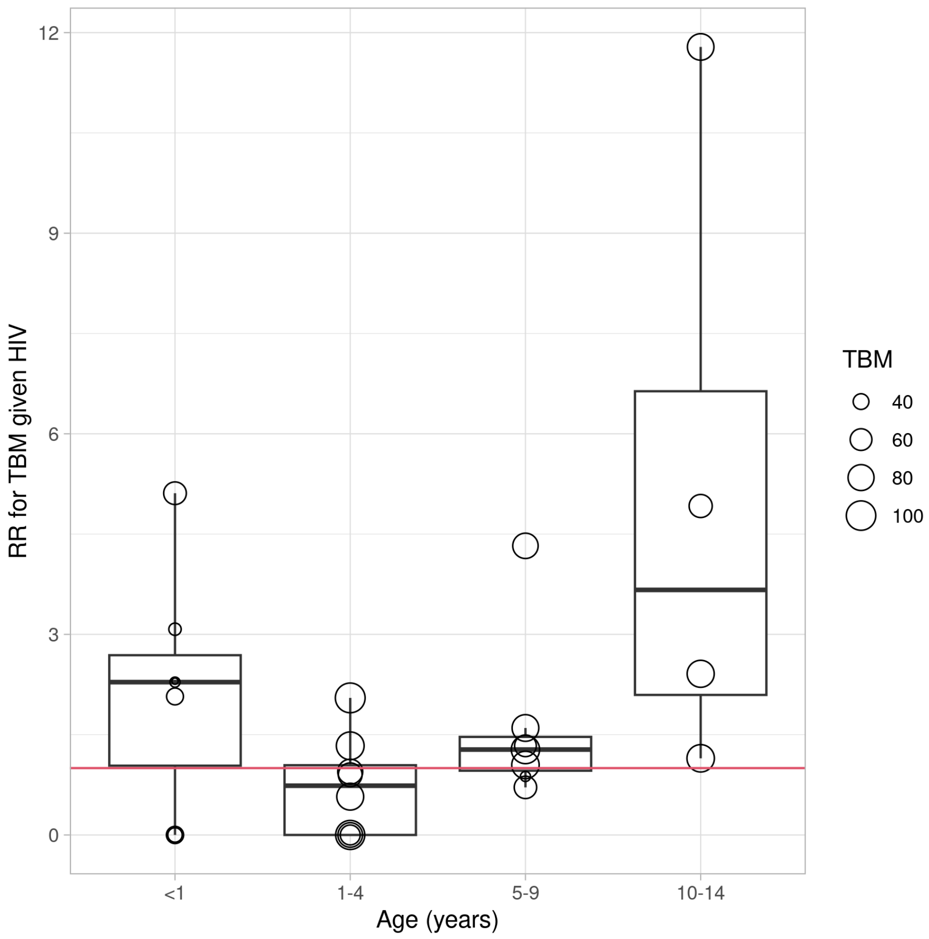

Figure A8 Empirical risk ratios for death given HIV infection among notified TBM in South Africa

Because we lack age-specific prevalence data on HIV, we generated only an age-independent relative risk of death given HIV. We performed a random effects meta-analysis of the South African data (Figure A8) to produce an age-independent odds ratio of tuberculosis deaths being TBM given HIV: 2.07 (95%CI 1.45 to 2.95).

Notification splits

Notification age-split

Since WHO notification data are split only into 0-4 and 5-14 year age groups, we conducted a random effects binomial meta-analysis of individual country surveillance data to estimate the proportion of TBM notifications in 0-4 year-olds that is among <1 year olds and the proportion of TBM notifications in 5-14 year-olds that is among 5-9 year olds, following the same two-step approach as above (see Figure A9 and Table A7).

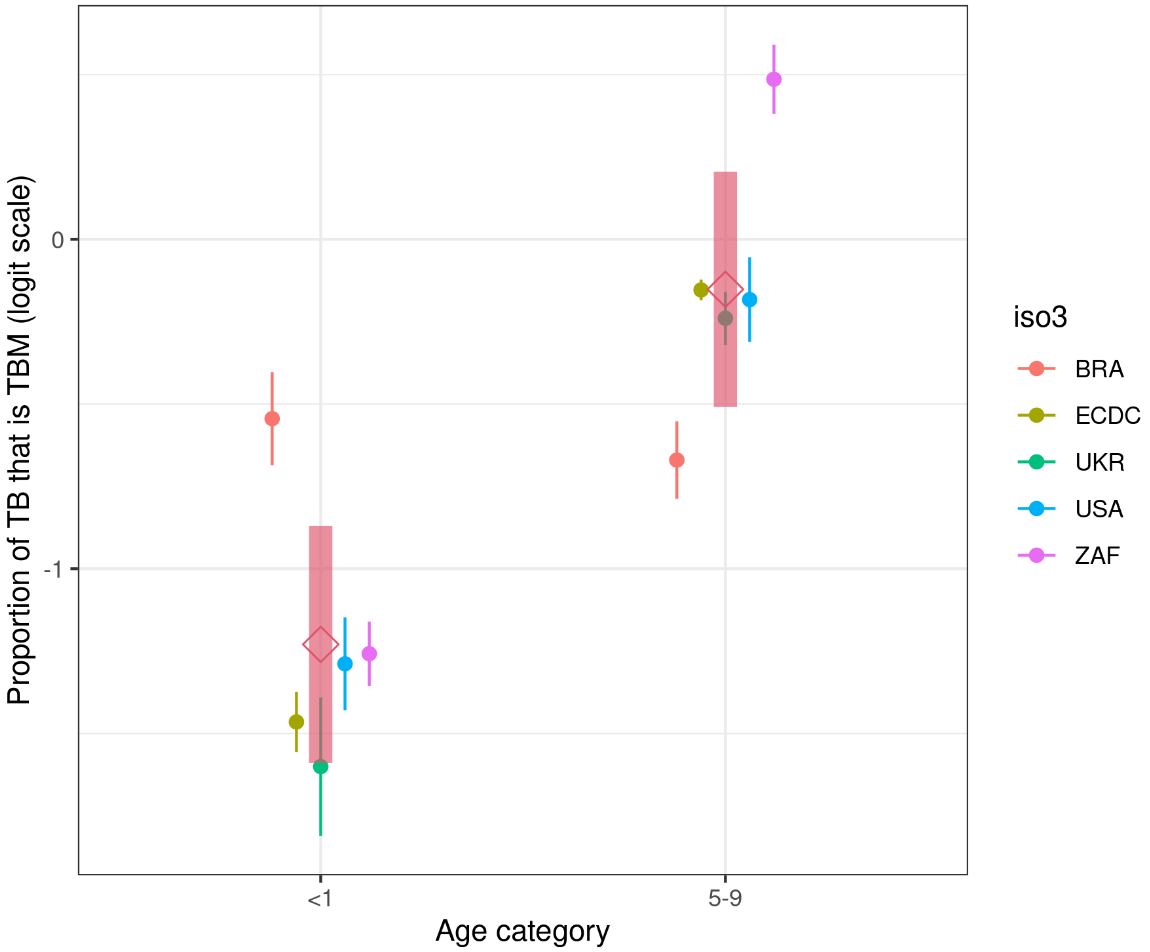

Figure A9 Meta-analysis of the proportion of TBM notifications in 0-4 and 5-14 year age groups in youngest subgroup
Note: Red diamonds represent pooled estimates

Table A7 Meta-analytic estimates tuberculosis notifications proportions in youngest subgroups

Age category (years)	Proportion	Lower 95% CI of proportion	Upper 95% CI of proportion
<1	22.63%	16.94%	29.53%
5-9	46.21%	37.54%	55.12%

Notification HIV splits

Paediatric estimates of the prevalence of HIV among notified tuberculosis are not routinely produced, but WHO do estimate the proportion of tuberculosis incidence among people living with HIV (aggregated across all ages). We used modelling estimates of the proportion of tuberculosis disease in children aged 0-14 years living with HIV from Dodd et al.[16] as the basis for a rule to convert the WHO all-age estimate to a paediatric specific estimate. We used linear regression of the WHO all-age HIV-in-tuberculosis estimates for 2015 (Figure A10).

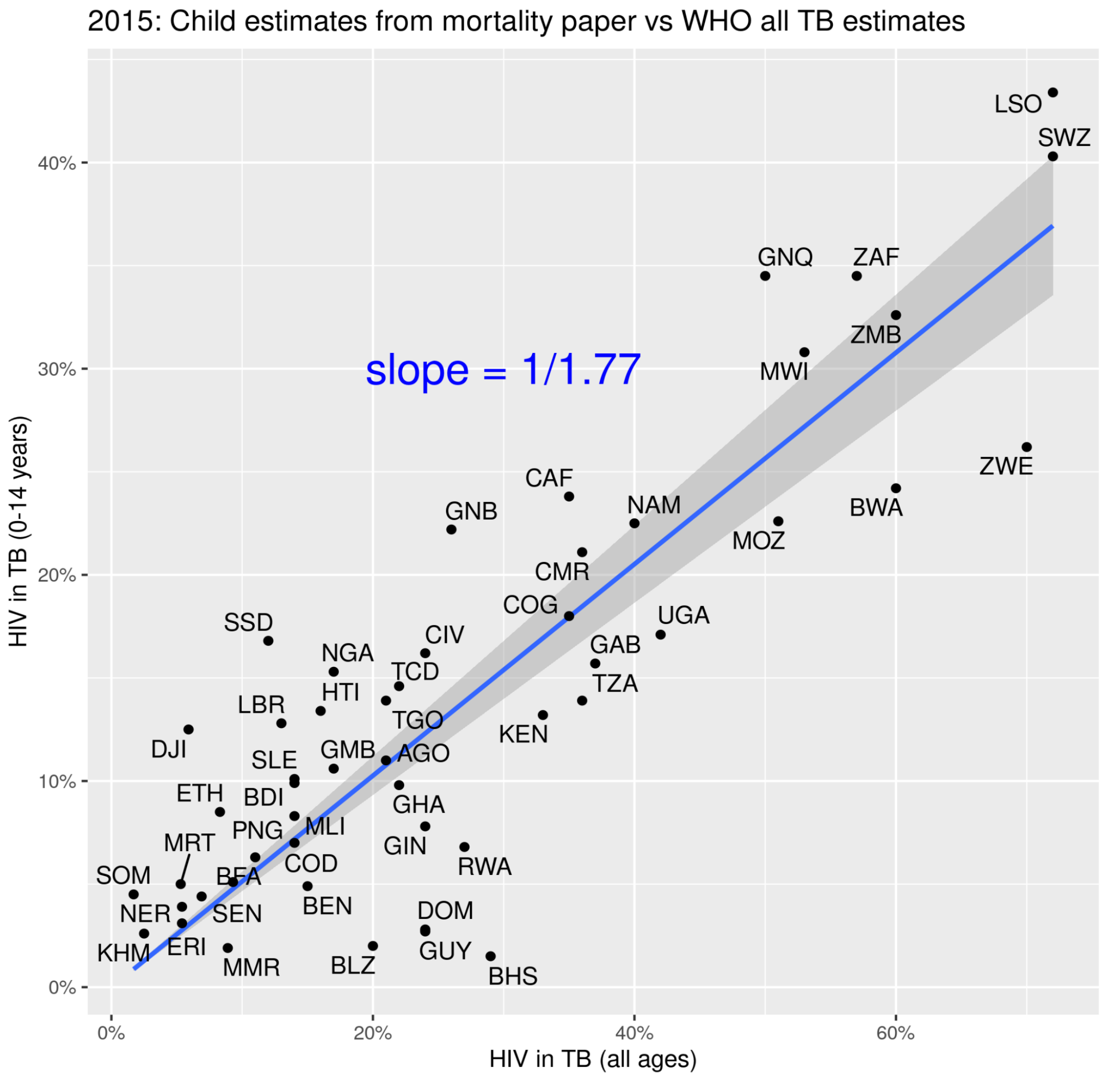

Figure A10 Relation between all-age HIV-in-tuberculosis and HIV-in-tuberculosis for 0–14-year-olds

Mathematical modelling of TBM incidence, morbidity, and mortality

Other input data sources & preparation

WHO notification data for 2019 and estimates of HIV prevalence in tuberculosis, and estimates of tuberculosis incidence by country, sex and for ages 0-4 and 5-14 years were obtained from https://www.who.int/teams/global-tuberculosis-programme/data on 29/03/2023.

Input data on country BCG coverage in 2019 were from the WHO/UNICEF estimates of national immunization coverage (WUENIC) on 26/09/2022 (the 14/07/2020 data update), now available at https://immunizationdata.who.int/ Where country estimates were missing, we used the global mean coverage.

Estimates of tuberculosis prevalence and population size and structure in 2019 were taken from Institute of Health Metrics and Evaluation (IHME) analyses available 24/10/2022 via the GBD results tool at https://vizhub.healthdata.org/gbd-results/

After merging, we were able to consider 202 countries, namely those with ISO3 codes:

AFG AGO ALB AND ARE ARG ARM ASM ATG AUS AUT AZE BDI BEL BEN BFA BGD BGR BHR BHS BIH BLR BLZ BMU BOL BRA BRB BRN BTN BWA CAF CAN CHE CHL CHN CIV CMR COD COG COK COL COM CPV CRI CUB CYP CZE DEU DJI DMA DNK DOM DZA ECU EGY ERI ESP EST ETH FIN FJI FRA FSM GAB GBR GEO GHA GIN GMB GNB GNQ GRC GRD GRL GTM GUM GUY HND HRV HTI HUN IDN IND IRL IRN IRQ ISL ISR ITA JAM JOR JPN KAZ KEN KGZ KHM KIR KNA KOR KWT LAO LBN LBR LBY LCA LKA LSO LTU LUX LVA MAR MCO MDA MDG MDV MEX MHL MKD MLI MLT MMR MNE MNG MNP MOZ MRT MUS MWI MYS NAM NER NGA NIC NIU NLD NOR NPL NRU NZL OMN PAK PAN PER PHL PLW PNG POL PRI PRK PRT PRY PSE QAT ROU RUS RWA SAU SDN SEN SGP SLB SLE SLV SMR SOM SRB SSD STP SUR SVK SVN SWE SWZ SYC SYR TCD TGO THA TJK TKL TKM TLS TON TTO TUN TUR TUV TZA UGA UKR URY USA UZB VCT VEN VNM VUT WSM YEM ZAF ZMB ZWE

Modelling approach

Our approach was to combine the country-level input data described above with results from literature and our meta-analyses above to model priors for the TBM incidence and notifications by age in each country (see Figure 2 in main paper). We combined these estimates using a model of the case detection ratio (CDR) as the likelihood, constructed from the WHO age-specific CDR estimates. We sampled from the resulting joint distribution with Markov chain Monte Carlo (MCMC) methods using Stan (version 2.23.2). Uncertainty was included via prior distributions defined in Table A8, except for the CDR, which defined the likelihood. Country-specific distributions were defined to match mean and standard deviation of corresponding quantities.

The prior for TBM incidence $I_{c,a}$ in country c and age group a was:

$$I_{c,a} = P_{c,a}\lambda_c(1 - (1 - V)C_c)\pi_a$$

where $P_{c,a}$ is the corresponding population size, λ_c is the force of tuberculosis infection estimated from tuberculosis prevalence via Styblo's rule, V is the protection against TBM afforded by BCG vaccination as a hazard ratio and C_c the coverage. Finally, π_a is the progression risk from infection to incident TBM from analyses above.

The prior for TBM notifications $N_{c,a}$ in country c and age group a was:

$$N_{c,a} = N_{c,A(a)} f_a \phi_{c,a}$$

where $N_{c,A(a)}$ is the reported number of tuberculosis notifications for the aggregate age group $A(a)$ containing a, f_a is the fraction of notifications in age group $A(a)$ estimated to be in subgroup a from our analysis above, and $\phi_{c,a}$ is the fraction of tuberculosis notifications in country c and age group a estimated to be TBM. This fraction was modelled as $\phi_{c,a} = (1 - h_c)\phi_a + h_c\widetilde{\phi_a}$ where h_c is the estimated HIV prevalence in incident tuberculosis for paediatric age groups (see above), ϕ_a is the estimated fraction of incident tuberculosis that is TBM by age from above, and $\widetilde{\phi_a}$ is this fraction with the odds ratio for TBM in incident tuberculosis given HIV infection from above applied.

The log-likelihood (LL) to combine the progression-based and notification-based TBM estimates was

$$LL = -\sum \frac{\left(\frac{N_{c,a}}{I_{c,a}} - \mu_{c,A(a)}\right)^2}{2\sigma_{c,A(a)}}$$

where the sum is over countries and age groups, and $\mu_{c,A(a)}$ and $\sigma_{c,A(a)}$ are the mean and standard deviation of each country's WHO estimated CDR, respectively, for the aggregate age group $A(a)$ containing age group a.

Related quantities were calculated within the MCMC framework as Stan generated quantities. The HIV-associated TBM incidence was calculated as $I^+_{c,a} = H_{c,a}I_{c,a}$ where

$H_{c,a} = \frac{h_c\phi_a}{\phi_{c,a}}$

Deaths $D_{c,a}$ were estimated as

$$D_{c,a} = \left[I_{c,a} - N_{c,a}\right]_+ + N_{c,a}\left(\psi^-_a(1 - H_{c,a}) + \psi^+_a H_{c,a}\right)$$

where $\left[I_{c,a} - N_{c,a}\right]_+$ is untreated TBM where positive (i.e. zero when TBM estimated as higher than notifications), ψ^-_a is the case fatality ratio (CFR) in HIV-negative children with TBM who receive treatment modelled as described above, and ψ^+_a is the same CFR with the odds ratio for death on treatment if HIV-negative from above applied. Similarly, morbidity was estimated by applying the age-specific morbidity rate described above to those surviving TBM.

Regional, global, and other aggregates were also computed in Stan, and their uncertainty intervals computed from the chains directly as 95% quantiles. All parameters (except BCG coverage, which lacked uncertainty estimates) were treated as uncertain; the priors are described in Table A8. Inference

was based on 4 chains of length 5,000, with a burn-in of 2,500. Sample R-squared values of <1.002 for global quantities suggested adequate convergence, and effective sample sizes (ESS) for global TBM incidence and mortality exceeded 8,000.

Table A8 Table of priors LN=log-Normal; B=beta; parameters not listed are treated as deterministic

Parameter description	Distribution	Mean (IQR)	Source	Source type
Tuberculosis prevalence	country-specific log-normal	-	IHME	Country estimates
WHO CDR	country-specific normal likelihood contribution	-	WHO	
HIV in tuberculosis	country-specific log-normal	-	WHO adjusted	
Styblo ratio	LN(1.677891,0.3714703)	5.7 (4.2-6.9)	Dodd et al[17]	Literature
BCG protection HR	B(56.51845,152.8091)	27.0% (24.9%-29.0%)	Bourdin Trunz et al[18]	
CFR	LN(31.35629,131.1115)	19.3% (17.1%-21.3%)	Chiang et al.[15]	
TBM sequelae	B(40.8458,34.93491)	53.9% (50.0%-57.8%)	Chiang et al.[15]	
Notification age split, 0-4	LN(-1.2296044,0.1837043)	29.7% (25.8%-33.1%)	above	This analysis
Notification age split, 5-14	LN(-0.1518343,0.1837043)	87.4% (76.0%-97.1%)	above	
TBM risk, <1	LN(-2.124421,0.2508527)	12.3% (10.1%-14.2%)	above	
TBM risk,1-4	LN(-3.405414,0.1255827)	3.3% (3.0%- 3.6%)	above	
TBM risk, 5-9	LN(-4.762942,0.2280476)	0.9% (0.7%- 1.0%)	above	
TBM risk, 10-14	LN(-5.836077,0.5310728)	0.3% (0.2%- 0.4%)	above	
OR of TB notifications being TBM given HIV	LN(0.3087445,0.01722183)	1.4 (1.3-1.4)	above	
Proportion of tuberculosis notifications TBM, <1	LN(-3.064787,0.3197908)	4.9% (3.8%-5.8%)	above	
Proportion of tuberculosis notifications TBM, 1-4	LN(-3.437060,0.3068060)	3.4% (2.6%-4.0%)	above	
Proportion of tuberculosis notifications TBM, 5-9	LN(-4.087180,0.3197308)	1.8% (1.4%-2.1%)	above	
Proportion of tuberculosis notifications TBM, 10-14	LN(-4.305228,0.3207261)	1.4% (1.1%-1.7%)	above	
HIV CFR OR	LN(0.711144,0.03337776)	2.0 (2.0-2.1)	above	
CFR OR age, <1	LN(0.491827345,0.2460298)	1.7 (1.4-1.9)	above	
CFR OR age, 1-4	LN(0.056411727,0.1979469)	1.1 (0.9-1.2)	above	
CFR OR age, 5-9	LN(-0.009626139,0.2566902)	1.0 (0.8-1.2)	above	
CFR OR age, 10-14	LN(-0.712891449,0.3582677)	0.5 (0.4-0.6)	above	

Code and data availability

The code and data for the meta-analyses and the population estimates of TBM incidence, morbidity, and death are available at https://github.com/petedodd/TBMK

Acknowledgements

This study was funded by the Fogarty International Center of the US National Institutes of Health (award number K43TW011006), awarded to KdP. The content is solely the responsibility of the authors and does not necessarily represent the official views of the National Institutes of Health.

We thank Ben Marais and Peter Donald for their support in sourcing original papers from the pre-chemotherapy era and Adam Langer, Robert Pratt, Brittany Moore, and Julie Self from the US Centers for Disease Control and Prevention Division of Tuberculosis Elimination.

TB surveillance data from South Africa was based on routine data collected through the National TB Control Programme, Department of Health, South Africa.

Data from The European Surveillance System – TESSy, provided by Iceland, Malta, Luxembourg, Cyprus, Estonia, Slovenia, Croatia, Czechia, Hungary, Ireland, Norway, Denmark, Switzerland, Greece, Austria, Latvia, Slovakia, Sweden, Netherlands, Lithuania, Portugal, Belgium, Poland, Bulgaria, Germany, Italy, France, Spain, Romania and released by ECDC. The views and opinions of the authors expressed herein do not necessarily state or reflect those of ECDC. The accuracy of the authors' statistical analysis and the findings they report are not the responsibility of ECDC. ECDC is not responsible for conclusions or opinions drawn from the data provided. ECDC is not responsible for the correctness of the data and for data management, data merging and data collation after provision of the data. ECDC shall not be held liable for improper or incorrect use of the data.

Supplementary results

Table S1: Results stratified by tuberculosis treatment status, HIV, and age

Quantity	Tuberculosis treatment status	HIV	Age			
			<1	1-4	5-9	10-14
TBM incidence	untreated for tuberculosis	Not living with HIV	3 910 (2 770 to 5 050)	4 890 (3 640 to 6 130)	993 (307 to 1 680)	286 (0 to 682)
	untreated for tuberculosis	Living with HIV	358 (112 to 603)	445 (182 to 709)	126 (0 to 261)	34 (0 to 109)
	untreated for tuberculosis	Total	4 270 (3 100 to 5 430)	5 330 (4 060 to 6 600)	1 120 (420 to 1 820)	320 (0 to 723)
	treated for tuberculosis	Not living with HIV	3 620 (3 130 to 4 110)	5 000 (4 460 to 5 550)	2 720 (2 290 to 3 150)	859 (615 to 1 100)
	treated for tuberculosis	Living with HIV	228 (137 to 319)	321 (219 to 423)	161 (85 to 237)	51 (8.4 to 94)
	treated for tuberculosis	Total	3 850 (3 350 to 4 350)	5 330 (4 770 to 5 880)	2 880 (2 440 to 3 320)	910 (663 to 1 160)
TBM sequelae	untreated for tuberculosis	Not living with HIV	0 (0 to 0)	0 (0 to 0)	0 (0 to 0)	0 (0 to 0)
	untreated for tuberculosis	Living with HIV	0 (0 to 0)	0 (0 to 0)	0 (0 to 0)	0 (0 to 0)
	untreated for tuberculosis	Total	0 (0 to 0)	0 (0 to 0)	0 (0 to 0)	0 (0 to 0)
	treated for tuberculosis	Not living with HIV	1 600 (1 360 to 1 830)	2 110 (1 840 to 2 380)	1 150 (946 to 1 360)	359 (253 to 466)
	treated for tuberculosis	Living with HIV	101 (57 to 144)	136 (85 to 186)	68 (32 to 104)	21 (2.7 to 40)
	treated for tuberculosis	Total	1 700 (1 460 to 1 940)	2 250 (1 970 to 2 520)	1 220 (1 010 to 1 430)	381 (273 to 489)
TBM deaths	untreated for tuberculosis	Not living with HIV	3 920 (2 780 to 5 060)	4 910 (3 670 to 6 160)	990 (307 to 1 670)	286 (0 to 681)
	untreated for tuberculosis	Living with HIV	345 (99 to 591)	417 (156 to 678)	129 (0 to 274)	34 (0 to 114)
	untreated for tuberculosis	Total	4 270 (3 100 to 5 430)	5 330 (4 060 to 6 600)	1 120 (420 to 1 820)	320 (0 to 723)
	treated for tuberculosis	Not living with HIV	1 610 (227 to 3 000)	1 980 (483 to 3 470)	937 (210 to 1 660)	289 (0 to 707)
	treated for tuberculosis	Living with HIV	87 (0 to 396)	107 (0 to 430)	55 (0 to 216)	17 (0 to 104)
	treated for tuberculosis	Total	1 700 (280 to 3 120)	2 080 (555 to 3 610)	992 (248 to 1 740)	305 (0 to 732)

Table S2: Results stratified by tuberculosis treatment status, HIV, and WHO region[1]

Quantity	Tuberculosis treatment status	HIV	Africa	The Americas	Eastern Mediterranean	Europe	South-East Asia	Western Pacific	GLOBAL
TBM incidence	untreated for tuberculosis	Not living with HIV	5 680 (4 890 to 6 470)	181 (139 to 224)	1 130 (521 to 1 740)	113 (86 to 140)	1 960 (429 to 3 490)	1 000 (642 to 1 370)	10 200 (8 410 to 12 100)
	untreated for tuberculosis	Living with HIV	861 (554 to 1 170)	17 (4.2 to 30)	9 (0 to 65)	11 (2.8 to 20)	45 (0 to 276)	20 (0 to 71)	796 (285 to 1 310)
	untreated for tuberculosis	Total	6 550 (5 700 to 7 390)	198 (154 to 243)	1 140 (528 to 1 750)	124 (96 to 152)	2 010 (456 to 3 560)	1 020 (658 to 1 390)	11 000 (9 130 to 12 900)
	treated for tuberculosis	Not living with HIV	3 770 (3 490 to 4 060)	223 (203 to 242)	2 080 (1 730 to 2 430)	153 (139 to 168)	4 810 (4 070 to 5 550)	1 160 (992 to 1 340)	12 000 (11 200 to 12 900)
	treated for tuberculosis	Living with HIV	574 (464 to 684)	21 (15 to 27)	17 (0 to 49)	16 (11 to 20)	110 (0 to 221)	23 (0 to 47)	934 (693 to 1 170)
	treated for tuberculosis	Total	4 350 (4 040 to 4 650)	244 (223 to 264)	2 100 (1 740 to 2 450)	169 (154 to 184)	4 920 (4 170 to 5 670)	1 190 (1 010 to 1 360)	13 000 (12 100 to 13 900)
TBM sequelae	untreated for tuberculosis	Not living with HIV	0 (0 to 0)	0 (0 to 0)	0 (0 to 0)	0 (0 to 0)	0 (0 to 0)	0 (0 to 0)	0 (0 to 0)
	untreated for tuberculosis	Living with HIV	0 (0 to 0)	0 (0 to 0)	0 (0 to 0)	0 (0 to 0)	0 (0 to 0)	0 (0 to 0)	0 (0 to 0)
	untreated for tuberculosis	Total	0 (0 to 0)	0 (0 to 0)	0 (0 to 0)	0 (0 to 0)	0 (0 to 0)	0 (0 to 0)	0 (0 to 0)
	treated for tuberculosis	Not living with HIV	1 620 (1 480 to 1 750)	95 (86 to 105)	890 (720 to 1 060)	65 (59 to 72)	2 060 (1 700 to 2 410)	498 (414 to 582)	5 150 (4 730 to 5 560)
	treated for tuberculosis	Living with HIV	246 (192 to 299)	9.1 (6.2 to 12)	7.5 (0 to 23)	6.8 (4.5 to 9)	47 (0 to 101)	9.8 (0 to 22)	399 (283 to 515)
	treated for tuberculosis	Total	1 860 (1 710 to 2 010)	104 (94 to 114)	897 (727 to 1 070)	72 (65 to 79)	2 100 (1 740 to 2 460)	508 (423 to 593)	5 550 (5 110 to 5 980)
TBM deaths	untreated for tuberculosis	Not living with HIV	5 730 (4 940 to 6 530)	180 (137 to 222)	1 130 (520 to 1 740)	112 (85 to 139)	1 960 (427 to 3 490)	1 000 (640 to 1 360)	10 200 (8 390 to 12 100)
	untreated for tuberculosis	Living with HIV	812 (515 to 1 110)	19 (5.1 to 32)	11 (0 to 71)	12 (3.4 to 21)	49 (0 to 296)	21 (0 to 74)	816 (295 to 1 340)
	untreated for tuberculosis	Total	6 550 (5 700 to 7 390)	198 (154 to 243)	1 140 (528 to 1 750)	124 (96 to 152)	2 010 (456 to 3 560)	1 020 (658 to 1 390)	11 000 (9 130 to 12 900)

	treated for tuberculosis	Not living with HIV	1 220 (221 to 2 230)	72 (22 to 121)	739 (64 to 1 410)	57 (26 to 88)	2 280 (490 to 4 070)	441 (19 to 863)	4 700 (2 520 to 6 870)
	treated for tuberculosis	Living with HIV	174 (0 to 550)	7.6 (0 to 24)	7.4 (0 to 74)	6.6 (0 to 17)	60 (0 to 348)	9.9 (0 to 72)	385 (0 to 1 000)
	treated for tuberculosis	Total	1 400 (328 to 2 470)	79 (27 to 131)	746 (68 to 1 420)	64 (31 to 96)	2 340 (527 to 4 160)	451 (24 to 878)	5 080 (2 820 to 7 340)

[1]*Note: numbers are shown to 3 significant figures and so may not exactly aggregate to totals.*

References

1. Marais BJ, Gie RP, Schaaf HS, Hesseling AC, Obihara CC, Starke JJ, Enarson DA, Donald PR, Beyers N. The natural history of childhood intra-thoracic tuberculosis: a critical review of literature from the pre-chemotherapy era. Int J Tuberc Lung Dis. 2004;8(4):392-402. PubMed PMID: 15141729.
2. Wallgren A. Pulmonary tuberculosis—relation of childhood infection to disease in adults. Lancet. 1938;1:5973–6.
3. Brailey M. Prognosis in white and colored tuberculous children according to initial chest x-ray findings. Am J Public Health. 1943;33:343-52.
4. Davies PD. The natural history of tuberculosis in children. A study of child contacts in the Brompton Hospital Child Contact Clinic from 1930 to 1952. Tubercle. 1961;42(Suppl):1-40. PubMed PMID: 13720119.
5. Cammock RM, Miller FJ. Tuberculosis in young children. Lancet. 1953;1(6752):158-60. PubMed PMID: 13011997.
6. Payne M. Study of the natural history of primary tuberculosis in childhood with particular reference to the prevention of morbidity and mortality: University of Durham; 1959.
7. Miller FJW, Court SDM, Waltong WS, Knox EG. Growing up in Newcastle upon Tyne: Oxford University press; 1960.
8. Chu P, Chang Y, Zhang X, Han S, Jin Y, Yu Y, Yang Y, Feng G, Wang X, Shen Y, Ni X, Guo Y, Lu J. Epidemiology of extrapulmonary tuberculosis among pediatric inpatients in mainland China: a descriptive, multicenter study. Emerging microbes & infections. 2022;11(1):1090-102. Epub 2022/03/16. doi: 10.1080/22221751.2022.2054367. PubMed PMID: 35290153; PMCID: PMC9009909.
9. Donnan EJ, Coulter C, Simpson G, Clark J, Nourse C. Paediatric tuberculosis in Queensland, Australia: overrepresentation of cross-border and Indigenous children. Int J Tuberc Lung Dis. 2017;21(3):263-9. Epub 2017/02/23. doi: 10.5588/ijtld.16.0313. PubMed PMID: 28225336.
10. Ducomble T, Tolksdorf K, Karagiannis I, Hauer B, Brodhun B, Haas W, Fiebig L. The burden of extrapulmonary and meningitis tuberculosis: an investigation of national surveillance data, Germany, 2002 to 2009. Euro Surveill. 2013;18(12). PubMed PMID: 23557944.
11. Erkens CG, de Vries G, Keizer ST, Slump E, van den Hof S. The epidemiology of childhood tuberculosis in the Netherlands: still room for prevention. BMC Infect Dis. 2014;14:295. doi: 10.1186/1471-2334-14-295. PubMed PMID: 24885314; PMCID: PMC4068078.
12. Mohiyuddin T, Seddon JA, Thomas HL, Lalor MK. The Changing Landscape of Childhood Tuberculosis in the United Kingdom: A Retrospective Cohort (2000-2015). Pediatr Infect Dis J. 2019;38(5):470-5. Epub 2018/09/27. doi: 10.1097/inf.0000000000002200. PubMed PMID: 30256311.
13. Onyango DO, Yuen CM, Masini E, Borgdorff MW. Epidemiology of Pediatric Tuberculosis in Kenya and Risk Factors for Mortality during Treatment: A National Retrospective Cohort Study. J Pediatr. 2018;201:115-21. doi: 10.1016/j.jpeds.2018.05.017. PubMed PMID: 29885751.
14. Wang PD. Epidemiological trends of childhood tuberculosis in Taiwan, 1998-2005. Int J Tuberc Lung Dis. 2008;12(3):250-4. Epub 2008/02/21. PubMed PMID: 18284828.
15. Chiang SS, Khan FA, Milstein MB, Tolman AW, Benedetti A, Starke JR, Becerra MC. Treatment outcomes of childhood tuberculous meningitis: a systematic review and meta-analysis. Lancet Infect Dis. 2014;14(10):947-57. doi: 10.1016/S1473-3099(14)70852-7. PubMed PMID: 25108337.
16. Dodd PJ, Yuen CM, Sismanidis C, Seddon JA, Jenkins HE. The global burden of tuberculosis mortality in children: a mathematical modelling study. Lancet Glob Health. 2017;5(9):e898-e906. doi: 10.1016/S2214-109X(17)30289-9. PubMed PMID: 28807188; PMCID: PMC5556253.
17. Dodd PJ, Gardiner E, Coghlan R, Seddon JA. Burden of childhood tuberculosis in 22 high-burden countries: a mathematical modelling study. Lancet Glob Health. 2014;2(8):e453-9. doi: 10.1016/S2214-109X(14)70245-1. PubMed PMID: 25103518.

18. Trunz BB, Fine P, Dye C. Effect of BCG vaccination on childhood tuberculous meningitis and miliary tuberculosis worldwide: a meta-analysis and assessment of cost-effectiveness. Lancet. 2006;367(9517):1173-80. doi: 10.1016/S0140-6736(06)68507-3. PubMed PMID: 16616560.
